# Comparative Short-Term Electrochemical and Surface Characterization of Biodegradable Mg–Ca–Sr Alloys with Different Strontium Contents

**DOI:** 10.3390/jfb17080418

**Published:** 2026-08-20

**Authors:** Gabriela Leață, Dorin Ioan Cocoș, Ramona Feier, Corneliu Munteanu, Fabian Cezar Lupu, Ion Ciucă, Kamel Earar

**Affiliations:** 1 Medical and Pharmaceutical Research Center, Faculty of Medicine and Pharmacy, “Dunărea de Jos” University of Galati, 800008 Galati, Romania; gabrielaleata@yahoo.com (G.L.); kamel.earar@ugal.ro (K.E.); 2 Dental-Medicine Department, Faculty of Medicine and Pharmacy, “Dunărea de Jos” University of Galati, 800201 Galati, Romania; 3 Department of Dental Medicine, Dimitrie Cantemir University, 540545 Târgu Mureș, Romania; 4 Mechanical Engineering, Mechatronics and Robotics Department, “Gheorghe Asachi” Technical University of Iasi, 700050 Iasi, Romania; corneliu.munteanu@academic.tuiasi.ro (C.M.); fabian-cezar.lupu@academic.tuiasi.ro (F.C.L.); 5 Faculty Materials Science and Engineering, University Politehnica of Bucharest, 060042 Bucharest, Romania; ion.ciuca11@gmail.com

**Keywords:** biodegradable magnesium alloys, Mg–Ca–Sr alloys, strontium content, electrochemical corrosion, electrochemical impedance spectroscopy, Dulbecco’s phosphate-buffered saline

## Abstract

Biodegradable Mg–Ca–Sr alloys are promising candidates for temporary implant applications, but their degradation behavior is strongly influenced by alloy composition and electrolyte chemistry. This comparative short-term study investigated how increasing the Sr content from 0.5 to 1.5 wt.% influences the electrochemical response and surface characteristics of cast Mg–0.5Ca–xSr alloys in 0.9% NaCl and calcium- and magnesium-free Dulbecco’s phosphate-buffered saline (DPBS). Potentiodynamic polarization, electrochemical impedance spectroscopy, scanning electron microscopy, quantitative image analysis, and energy-dispersive X-ray spectroscopy were used. Increasing the Sr content reduced the corrosion-current density from 0.0110 to 0.0039 mA/cm^2^ in NaCl and from 0.297 to 0.169 mA/cm^2^ in DPBS, while the corresponding calculated corrosion rates decreased from 2.51 to 0.886 mm/year and from 6.66 to 3.79 mm/year, respectively. Replicated EIS measurements of both alloys showed that Mg–0.5Ca–1.5Sr exhibited higher charge-transfer resistance than Mg–0.5Ca–0.5Sr in both NaCl (253 ± 15 versus 184 ± 14 Ω·cm^2^) and DPBS (853 ± 48 versus 615 ± 42 Ω·cm^2^). NaCl-exposed surfaces showed more porous and discontinuous deposits and a greater number of visible pit-like defects, whereas DPBS produced comparatively continuous P-containing deposits. Overall, increasing the Sr content to 1.5 wt.% was associated with lower polarization-derived global corrosion kinetics and a more resistive interfacial response, whereas electrolyte composition strongly influenced both interfacial impedance and surface-deposit morphology. These findings represent comparative short-term electrochemical and surface-characterization data obtained at 23 ± 1 °C and should not be interpreted as measures of long-term physiological degradation or in vivo performance.

## 1. Introduction

Biodegradable magnesium-based alloys are promising candidates for temporary medical implants because their relatively low elastic modulus may reduce stress shielding, while progressive degradation can eliminate the need for secondary removal procedures [1]. These advantages are particularly relevant in oral and maxillofacial surgery, where fixation devices, membranes, and temporary supports are required mainly during the early stages of bone healing [2]. However, the rapid and often non-uniform corrosion of magnesium may cause premature loss of mechanical integrity, hydrogen evolution, and local alkalization, thereby limiting predictable clinical performance [3].

In oral bone regeneration, magnesium-based membranes and fixation systems are being investigated as resorbable alternatives to conventional non-degradable devices [4]. Although current evidence supports their potential in bone surgery, degradation kinetics, local tissue response, and long-term safety remain critical considerations [5]. Alloying is therefore essential for balancing mechanical stability and corrosion resistance. Strontium can modify grain structure, secondary-phase distribution, and electrochemical behavior [6], while studies on Mg–Zr–Sr-based alloys have shown that Sr concentration and processing conditions influence mechanical properties, corrosion resistance, and biocompatibility [7,8].

The Mg–Ca–Sr system is particularly relevant for biomedical applications because calcium contributes to grain refinement and bone-related biological functions, whereas strontium may promote osteogenic activity while modifying degradation kinetics [9]. Magnesium alloys may additionally exhibit antibacterial effects through Mg dissolution and the associated increase in local pH, although this response depends strongly on alloy composition and testing conditions [10]. Recent Mg–Ca–Sr investigations have reported promising mechanical, corrosion, and biological characteristics, supporting further optimization of these alloys for temporary implants [11].

Reliable evaluation of biodegradable metallic materials requires electrochemical testing combined with surface and degradation-product characterization. Studies of Fe- and Zn-based degradable systems have similarly shown that corrosion behavior should be interpreted together with surface morphology and corrosion-product composition [12,13]. In magnesium alloys, manufacturing route, alloy chemistry, material form, and phase distribution may substantially affect degradation behavior [14,15,16]. Biological studies further indicate that Mg–Sr and Mg–Ca–Sr alloys can support mesenchymal stem-cell responses, although ion release and corrosion products remain composition-dependent [17]. Surface coatings are widely investigated to control magnesium degradation, emphasizing the need to establish the intrinsic corrosion behavior of uncoated alloys before protective modifications are applied [18].

Magnesium corrosion involves anodic dissolution, hydrogen evolution, local alkalization, and the formation of surface layers whose composition and protectiveness depend on microstructure and electrolyte chemistry [19]. In simulated physiological fluids, these layers may contain magnesium hydroxide, phosphates, carbonates, and calcium-containing deposits [20]. Electrolyte selection is therefore critical because chloride, phosphate, bicarbonate, calcium, and magnesium ions can alter corrosion kinetics and film development [21]. In this context, 0.9% NaCl provides a chloride-rich medium suitable for assessing active dissolution, whereas calcium- and magnesium-free DPBS introduces phosphate-buffered conditions that may promote compositionally distinct surface deposits. Mg–Ca alloys provide an established basis for biodegradable-material development [22], while Mg–Zn–Ca studies have shown that processing history and phase distribution strongly influence corrosion behavior [23]. Sr addition may further modify grain size, secondary-phase formation, mechanical performance, and degradation response [24,25,26]. However, the effect of increasing Sr content within a fixed cast Mg–0.5Ca matrix, particularly across chemically distinct electrolytes, remains insufficiently characterized through combined electrochemical and retained-surface analyses.

Therefore, this study aimed to determine how increasing Sr content from 0.5 to 1.5 wt.% influences the short-term electrochemical response and surface characteristics of cast Mg–0.5Ca–xSr alloys in 0.9% NaCl and calcium- and magnesium-free DPBS. We hypothesized that the higher-Sr alloy would exhibit lower polarization-derived global corrosion kinetics and a more resistive interfacial response, whereas electrolyte composition would influence both electrochemical behavior and surface-deposit morphology. The experimental framework combined replicated potentiodynamic polarization and electrochemical impedance spectroscopy measurements for both alloy compositions in both electrolytes, retained-layer SEM/EDS analysis, and descriptive quantification of visible surface defects. The study was designed as a controlled electrochemical and surface-characterization screening investigation rather than as an assessment of long-term biodegradation, physiological performance, or in vivo suitability.

## 2. Materials and Methods

### 2.1. Alloy Preparation and Sample Processing

Two experimental magnesium alloys with nominal compositions of Mg–0.5Ca–0.5Sr and Mg–0.5Ca–1.5Sr, expressed in wt.%, were investigated. The alloys were produced by gravity casting using commercially pure magnesium and Mg–Ca and Mg–Sr master alloys. The nominal purities of the starting materials were 99.9% for Mg, 99.5% for Ca, and 99.5% for Sr. Melting was performed in a steel crucible at 720 ± 10 °C using an electric resistance furnace (Nabertherm L 9/11, Nabertherm GmbH, Lilienthal, Germany) under a protective atmosphere of argon containing 0.5 vol.% SF_6_. The melt temperature was monitored using a K-type thermocouple connected to a dual-input digital thermometer (Fluke 52 II, Fluke Corporation, Everett, WA, USA). After complete homogenization, the melts were held at the processing temperature for 10 min and poured into a cylindrical steel mold preheated to 200 °C in a laboratory drying oven (Memmert UF55, Memmert GmbH + Co. KG, Schwabach, Germany). The principal alloy-production procedure was consistent with that previously reported for comparable Mg–Ca–Sr systems [9,11].

The chemical composition of each cast alloy was verified by optical emission spectroscopy (OES) using a FOUNDRY-MASTER Smart optical emission spectrometer (Hitachi High-Tech Analytical Science GmbH, Uedem, Germany). The measured compositions are presented in Table 1. The Ca and Sr contents differed by no more than 0.02 wt.% from their respective nominal values, confirming conformity with the target alloy compositions. Fe, Ni, Cu, Mn, and Si were not intentionally added as alloying elements. They were detected by OES as residual trace constituents originating from the commercial starting materials and/or the casting process and were reported to provide complete compositional characterization of the experimental alloys.

The specimens were not produced by powder compaction, pellet pressing, or resin encapsulation. Instead, solid specimens measuring 10 mm in diameter and 5 mm in thickness were sectioned from the central region of the gravity-cast ingots using a low-speed precision diamond saw (IsoMet 1000, Buehler, Lake Bluff, IL, USA) to minimize thermal and mechanical alteration. Surface preparation was performed using an automated metallographic grinding and polishing system (Tegramin-25, Struers, Ballerup, Denmark). The exposed surfaces were sequentially ground using silicon carbide abrasive papers with grit sizes of 320, 600, 800, 1200, and 2000, followed by final polishing with a 1 µm diamond suspension. The specimens were subsequently rinsed with deionized water, ultrasonically cleaned in ethanol for 10 min using an ultrasonic bath (Elmasonic S 30 H, Elma Schmidbauer GmbH, Singen, Germany), and dried under a stream of compressed air.

After surface preparation, each specimen was mounted in a chemically inert polytetrafluoroethylene holder for electrochemical testing, leaving a circular exposed surface area of 0.785 cm^2^. The remaining surfaces were electrically insulated from the electrolyte. All electrochemical measurements were performed on freshly prepared surfaces, and a new specimen was used for each independent experiment.

### 2.2. Electrolyte Media

Corrosion behavior was evaluated in two aqueous electrolyte media: 0.9 wt.% sodium chloride solution and calcium- and magnesium-free Dulbecco’s phosphate-buffered saline (DPBS). The saline solution was freshly prepared by dissolving analytical-grade NaCl (≥99.5% purity) in deionized water at a concentration of 9.0 g/L. Calcium- and magnesium-free DPBS was obtained from Gibco, Thermo Fisher Scientific (Waltham, MA, USA; catalogue no. 14190-094) and was used without further dilution.

According to the manufacturer’s formulation, the DPBS contained approximately 8.0 g/L NaCl, 0.20 g/L KCl, 0.20 g/L KH_2_PO_4_, and 1.15 g/L Na_2_HPO_4_ and contained no added Ca^2+^ or Mg^2+^. The absence of calcium was relevant for distinguishing phosphate-containing deposits formed through interactions with Mg and Sr released from the alloys from externally supplied calcium-phosphate species.

Before each experiment, the initial pH of the electrolyte was measured using a calibrated pH meter. The initial pH values were 6.80 ± 0.05 for 0.9% NaCl and 7.40 ± 0.05 for DPBS. No additional pH adjustment was performed.

A fresh 100 mL volume of electrolyte was used for each specimen, corresponding to a solution-volume-to-exposed-area ratio of approximately 127 mL/cm^2^, based on an exposed specimen area of 0.785 cm^2^. Electrolytes were not reused between measurements.

All electrochemical experiments were performed at 23 ± 1 °C under naturally aerated conditions. The solutions were equilibrated before specimen immersion, and evaporation was minimized by partially covering the cell while maintaining atmospheric contact. This temperature was selected for standardized comparison of alloy- and electrolyte-dependent effects. Accordingly, the electrochemical parameters were interpreted as comparative laboratory screening data rather than direct predictors of in vivo degradation.

### 2.3. Electrochemical Measurements

Electrochemical measurements were performed using an OrigaFlex OGF500 potentiostat/galvanostat equipped with an electrochemical impedance spectroscopy module and controlled using OrigaMaster 5 software (OrigaLys ElectroChem SAS, Rillieux-la-Pape, France). A conventional three-electrode glass cell was used. The Mg–Ca–Sr specimen served as the working electrode; a platinum plate with a surface area substantially larger than that of the working electrode was used as the counter electrode, and a saturated calomel electrode (SCE) was used as the reference electrode. All potentials reported in this study are expressed relative to the SCE.

For each measurement, the working electrode was immersed in 100 mL of freshly prepared electrolyte. The solution was continuously stirred at 300 rpm using a magnetic stirring system to maintain homogeneous mass-transfer conditions while remaining naturally aerated. Before polarization or impedance measurements, the open-circuit potential was monitored for 30 min. Stabilization was considered achieved when the potential variation was lower than 2 mV during the final 5 min of monitoring. OCP values were extracted at the beginning of immersion and after 5, 10, 20, and 30 min. For each alloy–electrolyte condition, the values obtained from three independent specimens were summarized as mean ± standard deviation. The overall potential shift, ΔOCP, was calculated as the OCP at 30 min minus the initial OCP. For each specimen, the final 5 min variation was calculated as the difference between the maximum and minimum OCP values recorded between 25 and 30 min, after which the results were summarized as mean ± standard deviation.

Potentiodynamic polarization curves were recorded from −150 mV to +150 mV relative to the stabilized open-circuit potential at a scan rate of 1 mV/s. Separate freshly prepared specimens were used for each polarization experiment to avoid interference from previous electrochemical perturbation. The corrosion potential (E_corr), corrosion current density (j_corr), anodic and cathodic Tafel slopes (β_a and β_c), and polarization resistance (R_p) were determined using OrigaMaster 5 software. Polarization resistance was obtained directly from the OrigaMaster fitting output based on the fitted polarization parameters.

For the potentiodynamic polarization experiments, the total electrolyte-exposure time was approximately 35 min, comprising the 30 min open-circuit stabilization period followed by an approximately 5 min polarization scan from −150 to +150 mV relative to the stabilized OCP at a scan rate of 1 mV/s. The polarization scan constituted an externally applied electrochemical perturbation and therefore did not represent an unperturbed immersion condition.

Tafel fitting was performed only over visually linear semilogarithmic regions, generally located approximately 50–100 mV from E_corr, while excluding the immediate vicinity of the corrosion potential and any portions showing marked curvature, current oscillations, or evidence of surface-film instability. The anodic and cathodic branches were fitted separately, and only regions displaying acceptable linearity were retained. The goodness of linear fitting was assessed using the coefficient of determination (R^2^). For each independent polarization curve, the fitted potential intervals and corresponding R^2^ values were recorded separately for the anodic and cathodic branches. Both branches were included only when each exhibited a sufficiently linear semilogarithmic region.

Because magnesium alloys may exhibit time-dependent surface-film evolution, hydrogen evolution, and the negative difference effect during anodic polarization, the resulting j_corr, Tafel slopes, polarization-resistance values, and calculated corrosion rates were interpreted as comparative polarization-derived estimates rather than as absolute measures of long-term degradation.

The corrosion rate was calculated from the corrosion current density using Equation (1):

(1) $$v_{corr}=\frac{K\;j_{corr}\;EW}{\rho }$$

where $v_{corr}$ is the corrosion rate expressed in mm/year, $K=3.27\times 10^{-3}$ mm·g·µA^−1^·cm^−1^·year^−1^ is the unit-conversion constant, $j_{corr}$ is expressed in µA/cm^2^, EW is the equivalent weight of the alloy expressed in g/equivalent, and ρ is the alloy density expressed in g/cm^3^.

The equivalent weight was calculated from the nominal alloy composition using Equation (2):

(2) $$EW={\left(\sum_{i}\frac{f_{i}n_{i}}{A_{i}}\right)}^{-1}$$

where $f_{i}$ is the mass fraction, $n_{i}$ is the assumed valence, and $A_{i}$ is the atomic weight of element i. Valence states of +2 were used for Mg, Ca, and Sr. The calculated equivalent weights were approximately 12.13 g/equivalent for Mg–0.5Ca–0.5Sr and 12.17 g/equivalent for Mg–0.5Ca–1.5Sr. Alloy densities of approximately 1.75 and 1.77 g/cm^3^, respectively, were used for the corrosion-rate calculations.

Electrochemical impedance spectroscopy was performed for both Mg–0.5Ca–0.5Sr and Mg–0.5Ca–1.5Sr alloys in 0.9% NaCl and calcium- and magnesium-free DPBS. Measurements were conducted at the stabilized open-circuit potential over a frequency range from 100 kHz to 0.01 Hz using a sinusoidal voltage perturbation of 10 mV rms. The EIS measurements were performed using freshly prepared specimens under the same electrolyte-volume, temperature, aeration, and stirring conditions used for the polarization experiments.

For the EIS experiments, the total electrolyte-exposure time was approximately 48 min, comprising the 30 min open-circuit stabilization period followed by an approximately 18 min impedance acquisition under the selected frequency range and instrumental settings. Although EIS was performed using a small-amplitude sinusoidal perturbation near the stabilized OCP, it remained an electrochemical measurement rather than an unperturbed immersion test.

The impedance spectra were analyzed using Zview software, version 4.0c (Scribner Associates, Southern Pines, NC, USA), which supports equivalent-circuit modeling and area-normalized impedance parameters. The experimental spectra were fitted using the equivalent circuit given in Equation (3):

(3) $$R_{s}\left[Q\left(R_{ct}\left(LR_{L}\right)\right)\right]$$

**Figure 1:**
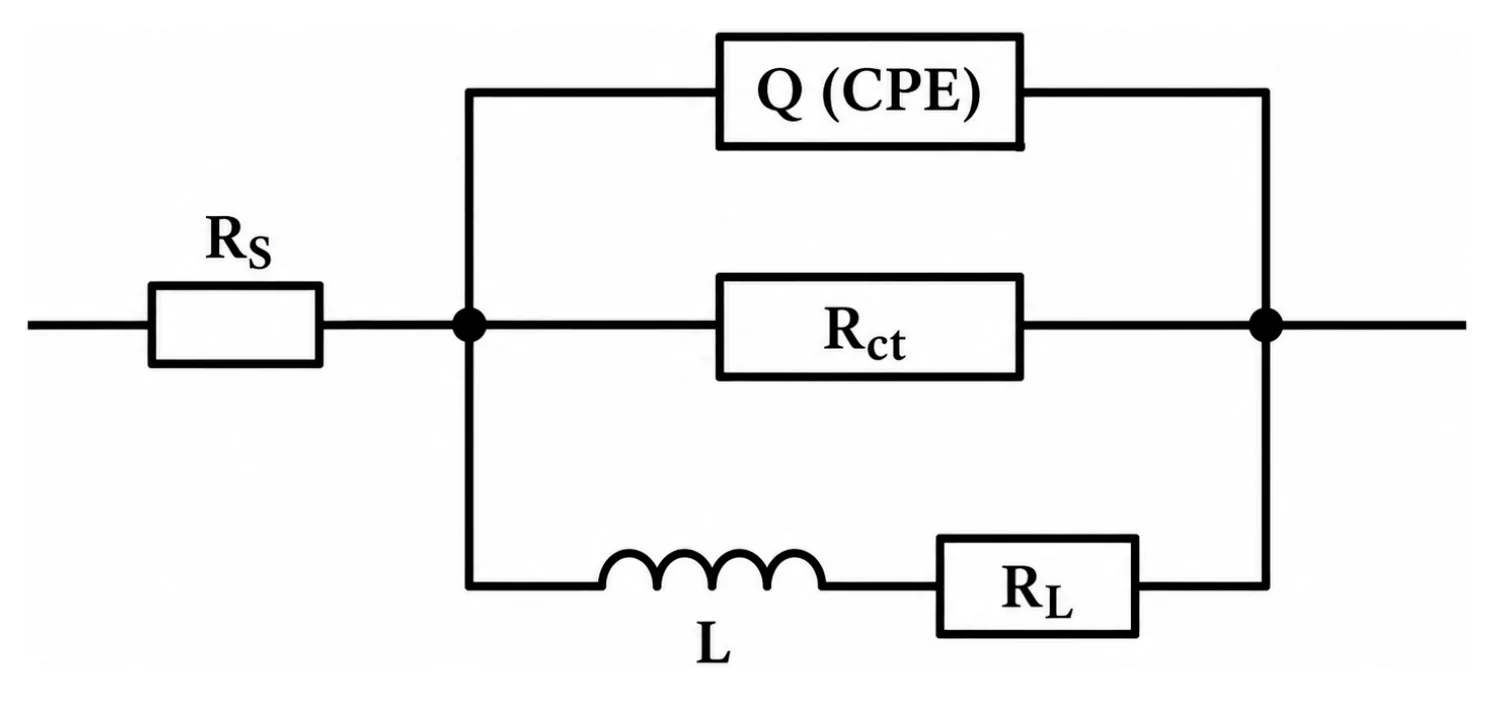
Schematic representation of the equivalent circuit $R_{s}\left[Q\left(R_{ct}\left(LR_{L}\right)\right)\right]$ used to fit the EIS spectra. $R_{s}$ represents the electrolyte resistance, Q is the constant-phase element, $R_{ct}$ is the charge-transfer resistance, L is the inductive element, and $R_{L}$ is the resistance associated with the inductive response.

A schematic representation of the equivalent circuit described by Equation (3) is shown in Figure 1. In this model, $R_{s}$ represents the electrolyte resistance, Q is the constant-phase-element coefficient associated with the non-ideal capacitive behavior of the corrosion-product layer, n is the corresponding CPE exponent, $R_{ct}$ is the charge-transfer resistance, L is the inductive element, and $R_{L}$ is the resistance associated with the inductive response. The impedance of the constant-phase element was defined using Equation (4):

(4) $$Z_{CPE}=\frac{1}{Q(j\omega {)}^{n}}$$

where j is the imaginary unit and ω is the angular frequency. The equivalent circuit was selected because it provided a physically plausible representation of the principal high- and low-frequency features observed in the impedance spectra. The constant-phase element was used to account for non-ideal capacitive behavior associated with surface heterogeneity and non-uniform corrosion-product coverage.

The low-frequency inductive branch was included phenomenologically to reproduce the observed response and may reflect transient adsorption–desorption or local formation and disruption of corrosion products. Because these processes were not independently verified, no unique physicochemical mechanism was assigned. Fit quality was assessed from agreement between experimental and fitted spectra, residual distributions, reduced χ^2^ values, and available parameter uncertainties.

### 2.4. SEM and EDS Characterization

Surface morphology and elemental composition were examined before and after electrochemical testing using a Thermo Scientific Quattro C scanning electron microscope (Thermo Fisher Scientific, Brno, Czech Republic) equipped with an integrated energy-dispersive X-ray spectroscopy system. Before corrosion testing, the polished alloy surfaces were examined to characterize the initial microstructural morphology and the distribution of secondary phases. After electrochemical exposure, the same analytical approach was used to evaluate corrosion-product morphology, visible localized surface defects, and changes in surface elemental composition.

Post-exposure SEM/EDS characterization was performed on specimens recovered immediately after the potentiodynamic polarization experiments. Accordingly, the analyzed surfaces had been exposed to the electrolyte for approximately 35 min, comprising the 30 min open-circuit stabilization period and the subsequent approximately 5 min polarization scan. The post-exposure micrographs therefore represent retained surface layers following short-term electrochemical polarization and should not be interpreted as the result of prolonged unperturbed immersion.

SEM images were acquired predominantly in secondary-electron mode using accelerating voltages of 15–20 kV, depending on the imaging and elemental-analysis requirements. The working distance ranged from approximately 9.5 to 14.1 mm, and beam spot sizes of 2.5–4.0 were used. Images were recorded at nominal magnifications ranging from 250× to 5000×. For direct comparison, all six overview micrographs were acquired at a nominal magnification of 750×, whereas the detailed morphological observations were acquired predominantly at 5000×. Low-magnification images were used to assess the overall distribution of secondary regions, corrosion products, and localized surface attack, whereas higher-magnification images were used to examine compositionally distinct secondary features, visible pit-like openings, microcavities, lamellar and granular deposits, porous structures, cracked layers, and other morphologically distinct corrosion-product regions. Backscattered-electron imaging was used for the initial alloy surfaces and selected compositionally distinct secondary regions, whereas secondary-electron imaging was used predominantly for the topographical characterization of the retained corrosion-product morphologies. Only global brightness and contrast adjustments were applied to improve visualization, without selective editing, removal, or artificial enhancement of individual surface features.

After electrochemical testing, the specimens were gently rinsed with deionized water to remove loosely adherent electrolyte residues without mechanically disturbing the corrosion-product layer. The surfaces were not chemically cleaned or etched. The specimens were subsequently dried under a stream of compressed air and stored in a desiccator until SEM examination. Consequently, the post-corrosion images represent the intact retained surface layer, including adherent corrosion products, rather than the morphology of the underlying metallic substrate after complete removal of these products.

Elemental analysis was performed using an Oxford Instruments X-MaxN 80 energy-dispersive X-ray detector (Oxford Instruments NanoAnalysis, High Wycombe, UK) integrated into the SEM system. EDS spectra and elemental maps were acquired at an accelerating voltage of 20 kV, with an acquisition time of 60 s per spectrum and a detector dead time maintained below 35%. The comparative spectra were acquired from the Mg–0.5Ca–1.5Sr alloy before testing and after exposure to 0.9% NaCl and DPBS using the same accelerating voltage, acquisition time, detector settings, and analyzed energy range. The spectra were independently normalized to their respective maximum intensities and displayed over the same energy range to facilitate qualitative comparison of the detected elemental signals. For each alloy–electrolyte condition, spectra were acquired from three selected surface regions exhibiting the characteristic corrosion morphology observed under that condition. The principal analyzed areas were approximately 100 × 100 µm, while smaller regions and individual morphologically distinct features were additionally selected for localized EDS analysis. These localized analyses included compositionally distinct secondary regions on the initial Mg–0.5Ca–1.5Sr surface and representative lamellar, granular, flake-like, compact, porous, and mixed deposits retained after electrochemical exposure.

Representative EDS elemental maps were acquired from selected surface fields of the Mg–0.5Ca–1.5Sr alloy before testing and after exposure to 0.9% NaCl and DPBS. For each mapped region, the SEM reference image and all corresponding elemental maps were obtained from the same field of view using identical spatial calibration and orientation. The mapped elements were selected according to the principal signals detected in the corresponding EDS spectra. The EDS results were treated as semi-quantitative estimates. Oxygen values, in particular, were used only for comparative surface characterization and were not interpreted as precise measurements of the oxygen content of the retained corrosion-product layers. Localized EDS analyses were used to characterize individual morphologically distinct regions, whereas the values represent the mean ± standard deviation obtained from three independently analyzed surface regions per alloy–electrolyte condition.

### 2.5. Descriptive Image-Based Surface Analysis

Descriptive image analysis was performed on selected SEM micrographs to estimate the apparent number, projected surface fraction, size, and morphology of visible localized surface defects after electrochemical exposure. Images were analyzed using Fiji/ImageJ software, version 1.54p (National Institutes of Health, Bethesda, MD, USA). Only micrographs acquired at identical magnification, pixel dimensions, and detector settings were compared directly.

Before segmentation, the images were converted to 8-bit grayscale and processed using a conservative workflow consisting of noise reduction, contrast-limited adaptive histogram equalization (CLAHE), and morphological filtering. Noise reduction was performed using a median filter with a radius of 1 pixel to suppress isolated high-frequency artifacts while preserving defect boundaries. Local contrast was enhanced using CLAHE with limited contrast amplification to minimize artificial enlargement of dark surface features.

Spatial calibration was performed using the scale bar embedded in each SEM micrograph. The resulting image resolution was approximately 0.11 µm/pixel. Surface defects were segmented using an adaptive local-thresholding procedure, followed by binary opening and closing operations to remove isolated pixels, fill minor discontinuities, and improve the continuity of the detected boundaries. The same processing parameters were applied to images acquired under comparable experimental conditions.

Objects touching the image borders were excluded to avoid incomplete measurements. Features smaller than 0.20 µm^2^ were excluded as probable image noise or superficial texture unrelated to clearly distinguishable surface cavities. Individual segmented objects were treated as visible pit-like openings or localized surface cavities and analyzed using the ImageJ particle-analysis module, version 1.54p (National Institutes of Health, Bethesda, MD, USA).

The following parameters were calculated:

- total number of detected visible defects or cavities;
- visible-defect density, expressed as defects/mm^2^;
- affected surface fraction, expressed as the percentage of the analyzed field;
- equivalent circular diameter;
- circularity;
- eccentricity.

The equivalent circular diameter was calculated from the measured object area using Equation (5):

(5) $$d_{eq}=2\sqrt{\frac{A}{\pi }}$$

where A is the projected area of the segmented defect. Circularity was calculated using Equation (6):

(6) $$C=\frac{4\pi A}{P^{2}}$$

where P is the perimeter of the object. Circularity values approaching 1 indicate a nearly circular projected geometry, whereas lower values indicate increasingly irregular shapes.

For each alloy–electrolyte condition, one SEM field was selected for descriptive analysis. The analyzed field was chosen from the low-magnification micrographs as an area displaying the characteristic surface morphology observed for that condition, while avoiding image borders, charging artifacts, and preparation-related defects. Because field selection was not randomized, only one image was analyzed per condition, and adherent corrosion products were retained, the resulting measurements were treated strictly as image-specific descriptive estimates of visible surface openings. They were not considered representative of the entire specimen surface, were not used for inferential statistical comparisons, and were interpreted together with the qualitative SEM morphology, EDS findings, and electrochemical measurements.

### 2.6. Replicates and Statistical Analysis

Open-circuit potential monitoring, potentiodynamic polarization, and electrochemical impedance spectroscopy measurements were independently repeated three times for each alloy–electrolyte combination using freshly prepared specimens. OCP values, overall potential shifts (ΔOCP), final 5 min potential variations, and polarization-derived electrochemical parameters, including *E*_corr_, *j*_corr_, *R*_p_, *β*_a_, *β*_c_, and *v*_corr_, are reported as mean ± standard deviation. The equivalent-circuit parameters obtained from EIS fitting, including $R_{s}$, Q, n, $R_{ct}$, L, and $R_{L}$, are also reported as mean ± standard deviation. Each EIS spectrum was fitted independently using the equivalent-circuit model described by Equation (3), after which the mean and standard deviation were calculated from the three independently fitted spectra for each alloy–electrolyte combination.

The effects of Sr content and electrolyte type were assessed using two-way ANOVA, followed by Tukey’s multiple-comparison test when significant main effects or interactions were identified. Residual normality and homogeneity of variances were evaluated using the Shapiro–Wilk and Levene tests, respectively. When these assumptions were not met, positively skewed variables such as *j*_corr_ and *v*_corr_ were logarithmically transformed. If the assumptions remained unsatisfied after transformation, the Kruskal–Wallis test followed by Dunn’s multiple-comparison procedure with adjusted *p*-values was used. Statistical significance was set at a two-sided *p* < 0.05, and exact *p*-values were reported whenever possible. Analyses were performed using GraphPad Prism version 10.4.1.

For the present dataset, the assumptions required for two-way ANOVA were considered acceptable for all analyzed electrochemical outcomes; therefore, analyses were performed on the original measurement scale, without logarithmic transformation or recourse to non-parametric testing.

The EIS measurements were performed for both Mg–0.5Ca–0.5Sr and Mg–0.5Ca–1.5Sr in both electrolytes. The impedance results were therefore used to compare the effects of Sr content and electrolyte composition on the interfacial electrochemical response. The EIS curves are representative of the three independent measurements obtained for each alloy–electrolyte combination, whereas the fitted parameters represent mean ± standard deviation from the three independently fitted spectra.

Similarly, the SEM image-analysis data were obtained from one selected field per experimental condition and were treated as descriptive, image-specific estimates. Defect counts, densities, affected surface fractions, and morphological descriptors were neither subjected to inferential statistical analysis nor extrapolated to the entire specimen surface.

Accordingly, the strength of inference differed among the analytical components. Replicated potentiodynamic polarization measurements were used for inferential statistical comparisons of alloy-composition and electrolyte effects, whereas replicated EIS measurements were used to compare the corresponding interfacial impedance responses. SEM/EDS findings and image-based surface measurements were treated as condition-specific descriptive observations. None of these analytical components was intended to quantify long-term physiological degradation or in vivo performance.

## 3. Results

### 3.1. Open-Circuit Potential Evolution

The open-circuit potential evolution recorded during the 30 min immersion period is summarized in Table 2. All alloy–electrolyte combinations exhibited an initial potential transient followed by progressive stabilization before the subsequent electrochemical measurements.

In 0.9% NaCl, the OCP of the Mg–0.5Ca–0.5Sr alloy shifted from −1568 ± 9 to −1539 ± 6 mV vs. SCE, corresponding to an overall positive displacement of 29 ± 5 mV. Under the same conditions, the OCP of the Mg–0.5Ca–1.5Sr alloy changed from −1557 ± 8 to −1529 ± 5 mV vs. SCE, with an overall shift of 28 ± 4 mV.

In calcium- and magnesium-free DPBS, the Mg–0.5Ca–0.5Sr alloy exhibited an OCP shift from −1679 ± 11 to −1645 ± 7 mV vs. SCE, whereas the Mg–0.5Ca–1.5Sr alloy changed from −1667 ± 10 to −1635 ± 7 mV vs. SCE. The corresponding overall potential shifts were 34 ± 6 and 32 ± 5 mV, respectively.

For all experimental conditions, the OCP shifted progressively in the positive direction, with most of the potential change occurring during the first 10–20 min of immersion. This behavior indicates an initial evolution of the alloy–electrolyte interface, likely associated with concurrent magnesium dissolution and the development of surface corrosion-product deposits. The OCP values recorded in DPBS remained more negative than those measured in 0.9% NaCl for both alloy compositions, demonstrating a marked electrolyte-dependent difference in the mixed electrochemical potential.

During the final 5 min of monitoring, the OCP variation remained below 2 mV for all alloy–electrolyte combinations, satisfying the predefined stabilization criterion before potentiodynamic polarization and impedance measurements. The OCP values were interpreted as indicators of the evolving electrochemical state of the alloy surface and were not considered direct measures of corrosion rate.

### 3.2. Potentiodynamic Polarization Behavior

**Figure 2:**
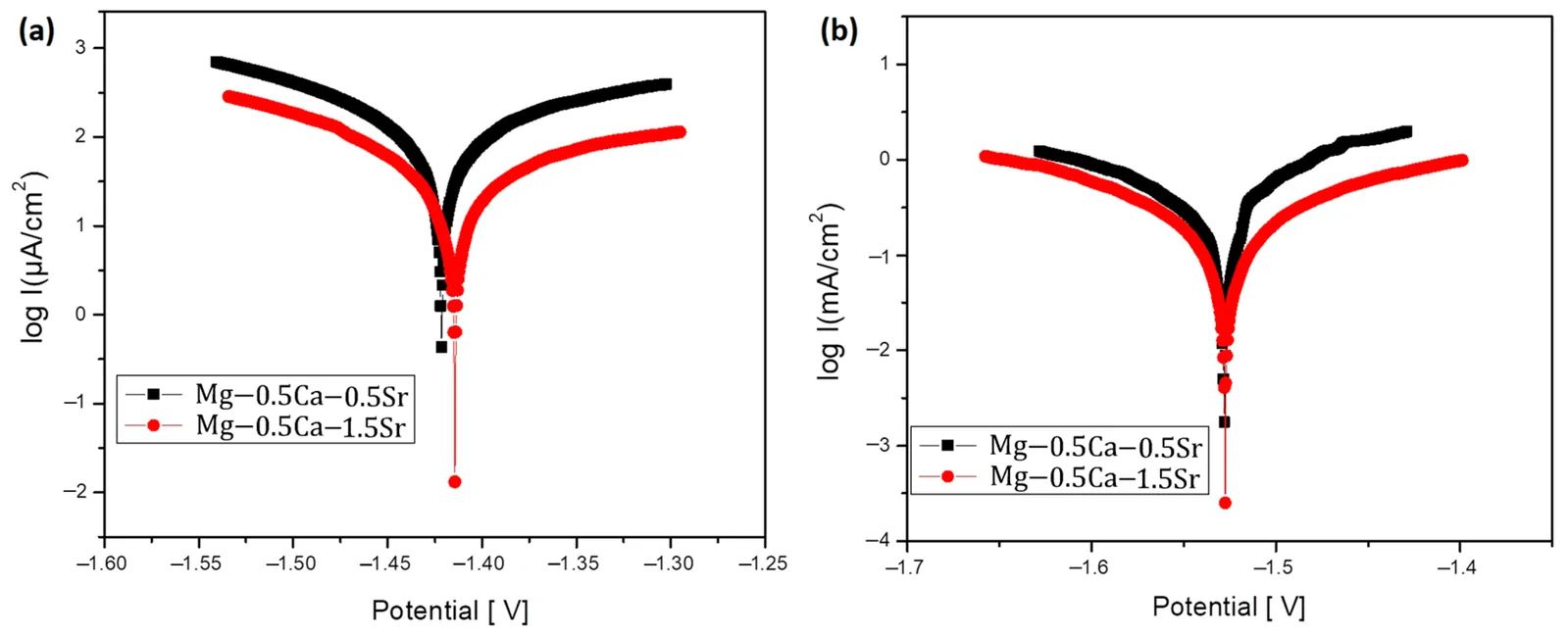
Representative potentiodynamic polarization curves of the Mg–0.5Ca–0.5Sr and Mg–0.5Ca–1.5Sr alloys in (**a**) 0.9% NaCl and (**b**) calcium- and magnesium-free Dulbecco’s phosphate-buffered saline (DPBS). Each curve is representative of three independent measurements performed for the corresponding alloy–electrolyte combination.

Representative potentiodynamic polarization curves of the Mg–0.5Ca–0.5Sr and Mg–0.5Ca–1.5Sr alloys recorded in 0.9% NaCl and calcium- and magnesium-free DPBS are presented in Figure 2. Both alloys exhibited a marked shift in their polarization response depending on the electrolyte medium. In DPBS, the curves were displaced toward higher current densities and more negative corrosion potentials compared with those recorded in NaCl. Within each medium, the alloy containing 1.5 wt.% Sr displayed lower corrosion-current densities than the alloy containing 0.5 wt.% Sr.

The electrochemical parameters obtained from Tafel analysis are summarized in Table 3. In 0.9% NaCl, increasing the Sr content from 0.5 to 1.5 wt.% reduced *j*_corr_ from 0.0110 ± 0.0014 to 0.0039 ± 0.0005 mA/cm^2^ and increased *R*_p_ from 239 ± 24 to 772 ± 68 Ω·cm^2^. The corresponding calculated corrosion rate decreased from 2.51 ± 0.32 to 0.886 ± 0.110 mm/year. A similar compositional effect was observed in DPBS, where the Mg–0.5Ca–1.5Sr alloy showed lower *j*_corr_, higher *R*_p_, and a lower calculated corrosion rate than the Mg–0.5Ca–0.5Sr alloy.

The assumptions of residual normality and homogeneity of variances were considered acceptable for all four electrochemical outcomes. Accordingly, $E_{corr}$, $j_{corr}$, $R_{p}$, and $v_{corr}$ were analyzed by two-way ANOVA on the original measurement scale. No logarithmic transformation or non-parametric analysis was required.

Two-way ANOVA showed a significant main effect of electrolyte medium on *E*_corr_ [*F*(1, 8) = 238.424, *p* < 0.0001], *j*_corr_ [*F*(1, 8) = 609.127, *p* < 0.0001], *R*_p_ [*F*(1, 8) = 384.143, *p* < 0.0001], and *v*_corr_ [*F*(1, 8) = 241.158, *p* < 0.0001]. Sr content significantly affected *j*_corr_ [*F*(1, 8) = 54.635, *p* < 0.0001], *R*_p_ [*F*(1, 8) = 192.984, *p* < 0.0001], and *v*_corr_ [*F*(1, 8) = 97.881, *p* < 0.0001], but did not significantly affect *E*_corr_ [*F*(1, 8) = 0.315, *p* = 0.5898]. Significant Sr-content-by-electrolyte interactions were identified for *j*_corr_ [*F*(1, 8) = 43.754, *p* = 0.0002], *R*_p_ [*F*(1, 8) = 126.569, *p* < 0.0001], and *v*_corr_ [*F*(1, 8) = 7.524, *p* = 0.0253], whereas the interaction was not significant for *E*_corr_ [*F*(1, 8) = 0.177, *p* = 0.6848]. Tukey-adjusted pairwise comparisons showed that Mg–0.5Ca–1.5Sr exhibited significantly lower *j*_corr_ and *v*_corr_ and significantly higher *R*_p_ than Mg–0.5Ca–0.5Sr in both electrolytes. Complete ANOVA results are provided in Supplementary Table S1.

Overall, DPBS produced higher corrosion-current densities and calculated corrosion rates than NaCl for both compositions. Nevertheless, increasing the Sr content from 0.5 to 1.5 wt.% consistently reduced the global corrosion kinetics under both experimental conditions.

### 3.3. Electrochemical Impedance Spectroscopy

**Figure 3:**
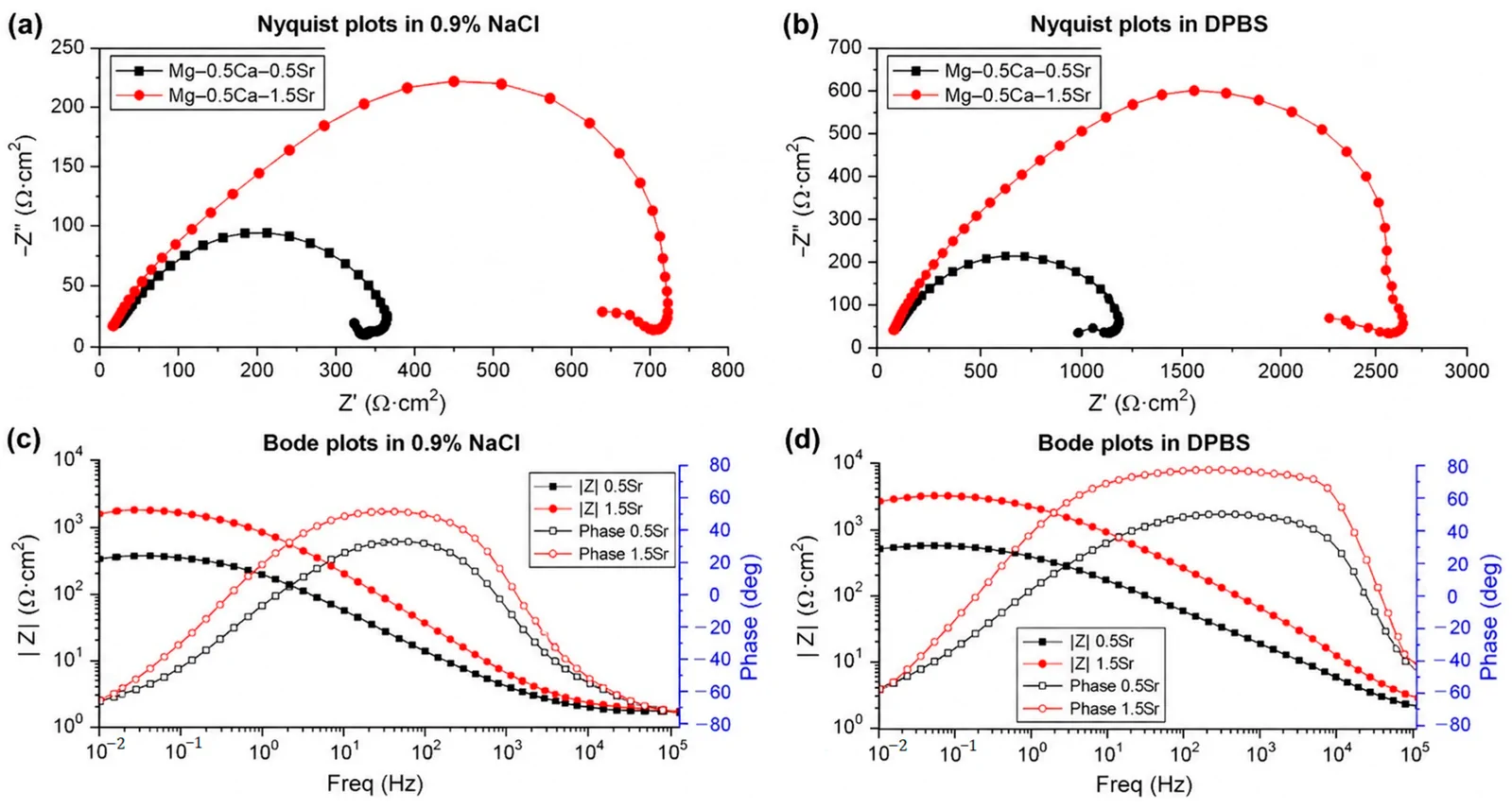
Electrochemical impedance spectroscopy responses of the Mg–0.5Ca–0.5Sr and Mg–0.5Ca–1.5Sr alloys in 0.9% NaCl and calcium- and magnesium-free Dulbecco’s phosphate-buffered saline (DPBS): (**a**) Nyquist plots in 0.9% NaCl; (**b**) Nyquist plots in DPBS; (**c**) combined Bode plots showing the impedance modulus, ∣Z∣, and phase angle in 0.9% NaCl; and (**d**) combined Bode plots showing the impedance modulus, ∣Z∣, and phase angle in DPBS. Symbols represent the experimental data, whereas the fitted curves correspond to the equivalent-circuit model described by Equation (3). Each curve is representative of three independent EIS measurements performed for the corresponding alloy–electrolyte combination.

Electrochemical impedance spectroscopy was performed for both Mg–0.5Ca–0.5Sr and Mg–0.5Ca–1.5Sr alloys in 0.9% NaCl and calcium- and magnesium-free DPBS to evaluate the effects of Sr content and electrolyte composition on the interfacial electrochemical response. The corresponding Nyquist and Bode plots are presented in Figure 3.

In 0.9% NaCl, the Mg–0.5Ca–1.5Sr alloy exhibited a larger capacitive loop and higher impedance-modulus values than the Mg–0.5Ca–0.5Sr alloy, indicating a more resistive alloy–electrolyte interface at the stabilized open-circuit potential. A similar compositional trend was observed in DPBS, where the Mg–0.5Ca–1.5Sr alloy also displayed a larger Nyquist semicircle and higher low-frequency impedance than the Mg–0.5Ca–0.5Sr alloy. For both compositions, the impedance response was more pronounced in DPBS than in NaCl, demonstrating that both Sr content and electrolyte composition influenced the interfacial electrochemical behavior under the tested conditions.

The phase-angle response further differentiated the alloy–electrolyte combinations. In 0.9% NaCl, the maximum absolute phase angle was approximately 35° for Mg–0.5Ca–0.5Sr and 40° for Mg–0.5Ca–1.5Sr. In DPBS, the corresponding maximum values were approximately 62° and 70°, respectively. The higher absolute phase angles observed for the Mg–0.5Ca–1.5Sr alloy indicate a more pronounced capacitive response, whereas the lower values recorded for Mg–0.5Ca–0.5Sr are consistent with a more heterogeneous and less ideally capacitive interface.

Nevertheless, the EIS results characterize the alloy–electrolyte interface under small-amplitude perturbation near the stabilized open-circuit potential and should not be interpreted as direct measures of the overall corrosion rate. The impedance findings were therefore interpreted together with the polarization, SEM, and EDS results.

The experimental spectra were fitted using the equivalent circuit $R_{s}\left[Q\left(R_{ct}\left(LR_{L}\right)\right)\right]$, comprising the solution resistance $R_{s}$, a constant-phase element Q, the charge-transfer resistance $R_{ct}$, and a low-frequency inductive branch containing L and $R_{L}$. The CPE accounted for non-ideal capacitive behavior, whereas the inductive branch was included phenomenologically to reproduce the low-frequency response.

As summarized in Table 4, in 0.9% NaCl, the Mg–0.5Ca–1.5Sr alloy exhibited a larger capacitive loop and a higher charge-transfer resistance than the Mg–0.5Ca–0.5Sr alloy, with $R_{ct}$ values of 253 ± 15 and 184 ± 14 Ω·cm^2^, respectively. A similar compositional trend was observed in DPBS, where $R_{ct}$ increased from 615 ± 42 Ω·cm^2^ for Mg–0.5Ca–0.5Sr to 853 ± 48 Ω·cm^2^ for Mg–0.5Ca–1.5Sr.

For both alloy compositions, DPBS produced higher charge-transfer resistance and lower Q values than NaCl, indicating a more resistive and compositionally distinct interface at the stabilized open-circuit potential. The CPE exponent remained below unity under all conditions, confirming non-ideal capacitive behavior associated with surface heterogeneity and non-uniform corrosion-product coverage.

The low-frequency inductive parameters also differed according to alloy composition and electrolyte type. DPBS produced markedly lower L and higher $R_{L}$ values than NaCl for both alloys, indicating distinct low-frequency interfacial processes. Reduced $\chi^{2}$ values were of the order of $10^{-4}$ for all conditions, indicating satisfactory agreement between the experimental and fitted spectra.

For both alloy compositions, DPBS produced higher interfacial impedance and higher $R_{ct}$ values than NaCl, despite the higher polarization-derived corrosion kinetics observed in DPBS. This divergence likely reflects the different conditions probed by the two techniques: EIS characterizes the interface near the stabilized open-circuit potential under small-amplitude perturbation, whereas potentiodynamic polarization progressively displaces the system from this quasi-steady state. Thus, the higher $R_{ct}$ values measured in DPBS indicate an apparently more resistive interface at the time of measurement, rather than sustained protection or lower overall degradation.

Within each electrolyte, the Mg–0.5Ca–1.5Sr alloy exhibited higher $R_{ct}$ values than the Mg–0.5Ca–0.5Sr alloy, indicating that Sr content also influenced the interfacial impedance response under the tested conditions. Nevertheless, these EIS findings should be interpreted together with the polarization, SEM, and EDS results and should not be regarded as direct measures of long-term corrosion resistance.

### 3.4. Alloy Surface Morphology Before and After Electrochemical Exposure

**Figure 4:**
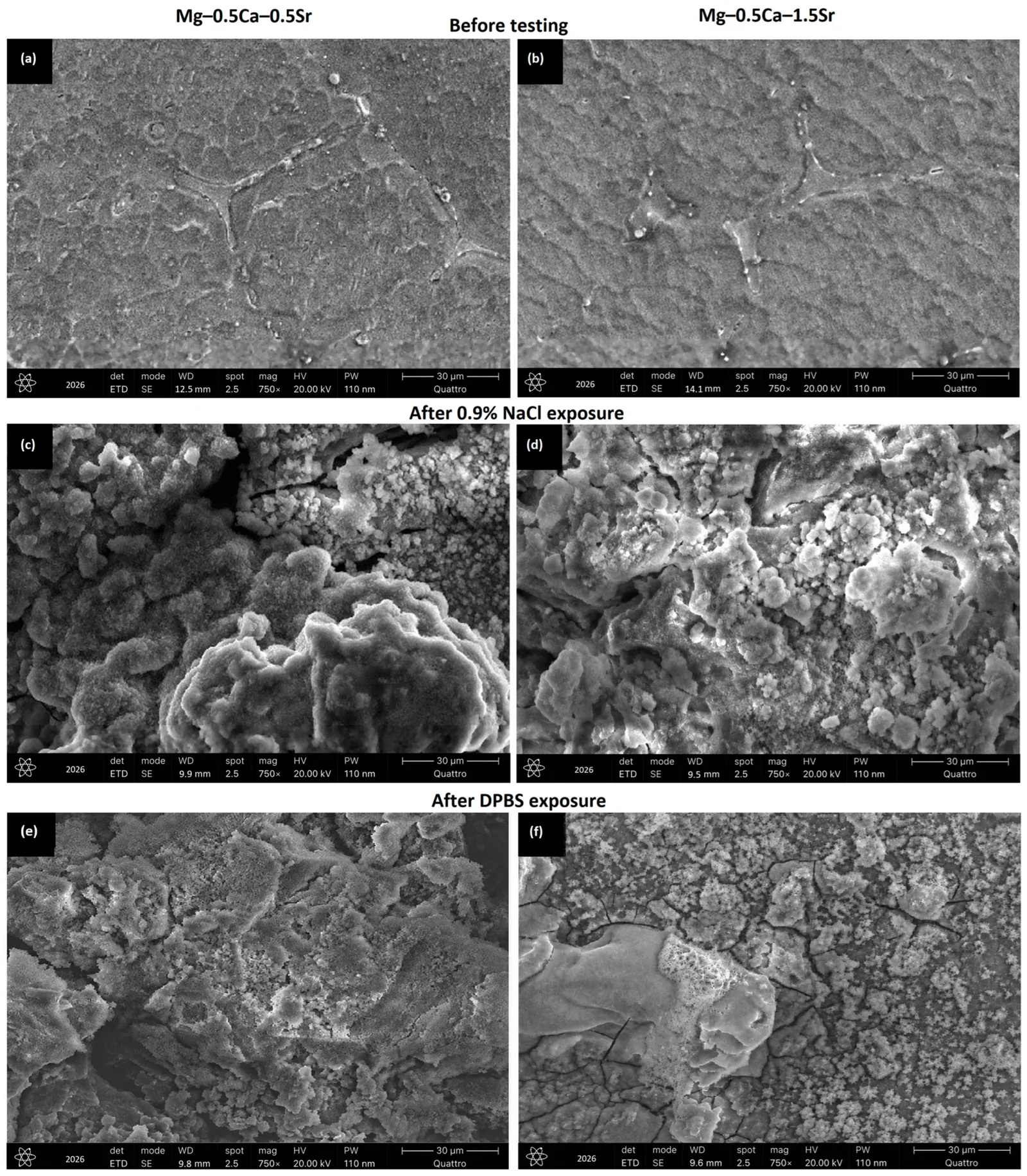
SEM micrographs of the experimental Mg–0.5Ca–0.5Sr and Mg–0.5Ca–1.5Sr alloys before and after electrochemical exposure. The successive rows show the surfaces before testing, after exposure to 0.9% NaCl, and after exposure to calcium- and magnesium-free Dulbecco’s phosphate-buffered saline (DPBS), respectively: (**a**) Mg–0.5Ca–0.5Sr before testing; (**b**) Mg–0.5Ca–1.5Sr before testing; (**c**) Mg–0.5Ca–0.5Sr after exposure to 0.9% NaCl; (**d**) Mg–0.5Ca–1.5Sr after exposure to 0.9% NaCl; (**e**) Mg–0.5Ca–0.5Sr after exposure to DPBS; and (**f**) Mg–0.5Ca–1.5Sr after exposure to DPBS. All images were acquired at 750× magnification. Scale bars: 30 µm.

The surface morphologies of the Mg–0.5Ca–0.5Sr and Mg–0.5Ca–1.5Sr alloys before and after electrochemical exposure are presented in Figure 4. The post-exposure specimens were examined with the corrosion-product layers retained; therefore, the micrographs show both adherent deposits and visible surface cavities rather than the fully exposed underlying metallic substrate.

The overall surface morphologies of both experimental alloys before and after electrochemical exposure are presented in Figure 4. Before electrochemical testing, the Mg–0.5Ca–0.5Sr alloy exhibited a comparatively homogeneous matrix containing relatively fine and discontinuously distributed brighter secondary regions, particularly along intergranular and interdendritic areas (Figure 4a). Wider matrix regions separated these brighter features.

The Mg–0.5Ca–1.5Sr alloy displayed a more pronounced and comparatively continuous network of brighter secondary regions (Figure 4b). These features were more extensively distributed along intergranular areas, while the intervening matrix regions appeared narrower than those observed in the alloy containing 0.5 wt.% Sr. Based on their morphology and compositional contrast, the brighter features were interpreted as Sr- and/or Ca-enriched secondary constituents morphologically compatible with intermetallic regions reported in comparable Mg–Ca–Sr systems. Their precise crystallographic identity could not be established by SEM alone.

After exposure to 0.9% NaCl, the Mg–0.5Ca–0.5Sr alloy surface was covered by a heterogeneous corrosion-product layer with a porous, granular, and locally agglomerated morphology (Figure 4c). Numerous microcavities and irregular depressions were visible between the corrosion-product aggregates. Localized surface attack was particularly apparent adjacent to compositionally distinct secondary regions and along portions of the intergranular relief.

The Mg–0.5Ca–1.5Sr alloy showed a similarly heterogeneous response in NaCl, although the corrosion-product aggregates appeared coarser and more extensively distributed across the analyzed field (Figure 4d). The surface contained irregular granular deposits, discontinuities, and visible cavity-like defects. The post-exposure morphology spatially followed the more pronounced intergranular distribution observed before testing.

For both compositions, the NaCl-exposed surfaces were characterized by discontinuous coverage, high apparent porosity, and clearly visible localized defects. Because the corrosion products were retained during SEM examination, the visible cavities should be interpreted as visible pit-like openings or surface depressions rather than as representations of the complete subsurface geometry of the corrosion damage.

Following exposure to DPBS, both alloys exhibited a surface morphology distinct from that observed after NaCl exposure. The Mg–0.5Ca–0.5Sr surface contained comparatively more continuous deposits and fewer clearly visible cavities within the analyzed field (Figure 4e). Several surface depressions appeared partially covered or filled by compact agglomerates, producing a less open and less porous appearance than the corresponding NaCl-exposed surface.

A similar response was observed for the Mg–0.5Ca–1.5Sr alloy (Figure 4f). The surface was covered by heterogeneous but comparatively continuous deposits, with localized compact regions superimposed on a finer granular background. Visible cavity openings were less prominent than in NaCl, and some defects appeared partially occluded by adherent corrosion products.

Overall, the SEM observations showed that the NaCl-exposed surfaces exhibited a more porous and discontinuous corrosion-product morphology with readily visible localized defects, whereas the DPBS-exposed surfaces showed comparatively more continuous coverage and partial occlusion of visible cavities. These differences were observed for both alloys, while the more pronounced initial intergranular heterogeneity remained evident in the Mg–0.5Ca–1.5Sr composition.

### 3.5. Descriptive Quantification of Visible Surface Defects

A descriptive image-based analysis was performed to estimate the number, density, surface fraction, size, circularity, and eccentricity of visible pit-like defects after electrochemical exposure. Because only one representative SEM field was analyzed per condition and corrosion products were retained, the results were treated as image-specific descriptive estimates of visible surface openings rather than statistically representative measurements of total corrosion damage.

As summarized in Table 5, the image-analysis values were obtained from the specific representative SEM fields shown in Figure 4c–f. The analyzed NaCl fields corresponded to the porous and granular morphologies shown in Figure 4c,d, whereas the analyzed DPBS fields corresponded to the comparatively continuous and partially occluding deposits shown in Figure 4e,f. The NaCl-exposed fields showed higher apparent defect counts, densities, and affected surface fractions than the corresponding DPBS-exposed fields for both alloys.

Within the analyzed fields, the NaCl-exposed surfaces showed a higher number, density, and affected surface fraction of visible defects than the corresponding DPBS-exposed fields. The visible defects detected after NaCl exposure also appeared less circular and more elongated than those identified after DPBS exposure. However, because the analysis was based on one representative field per condition and adherent corrosion products may partially obscure pit openings, these observations should not be extrapolated to the entire specimen surface or interpreted as statistically demonstrated differences.

### 3.6. Higher-Magnification Morphology and Localized Elemental Characterization

**Figure 5:**
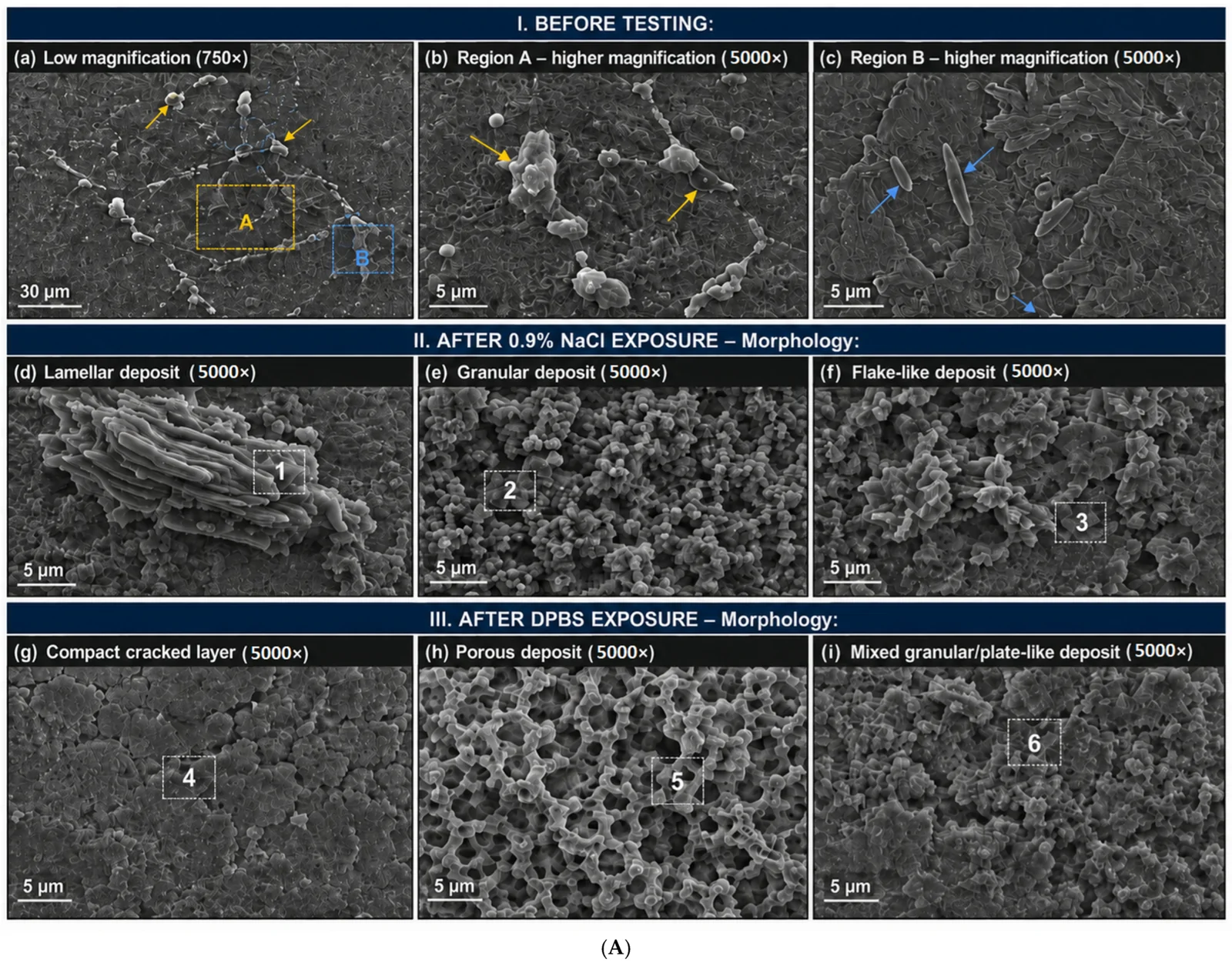

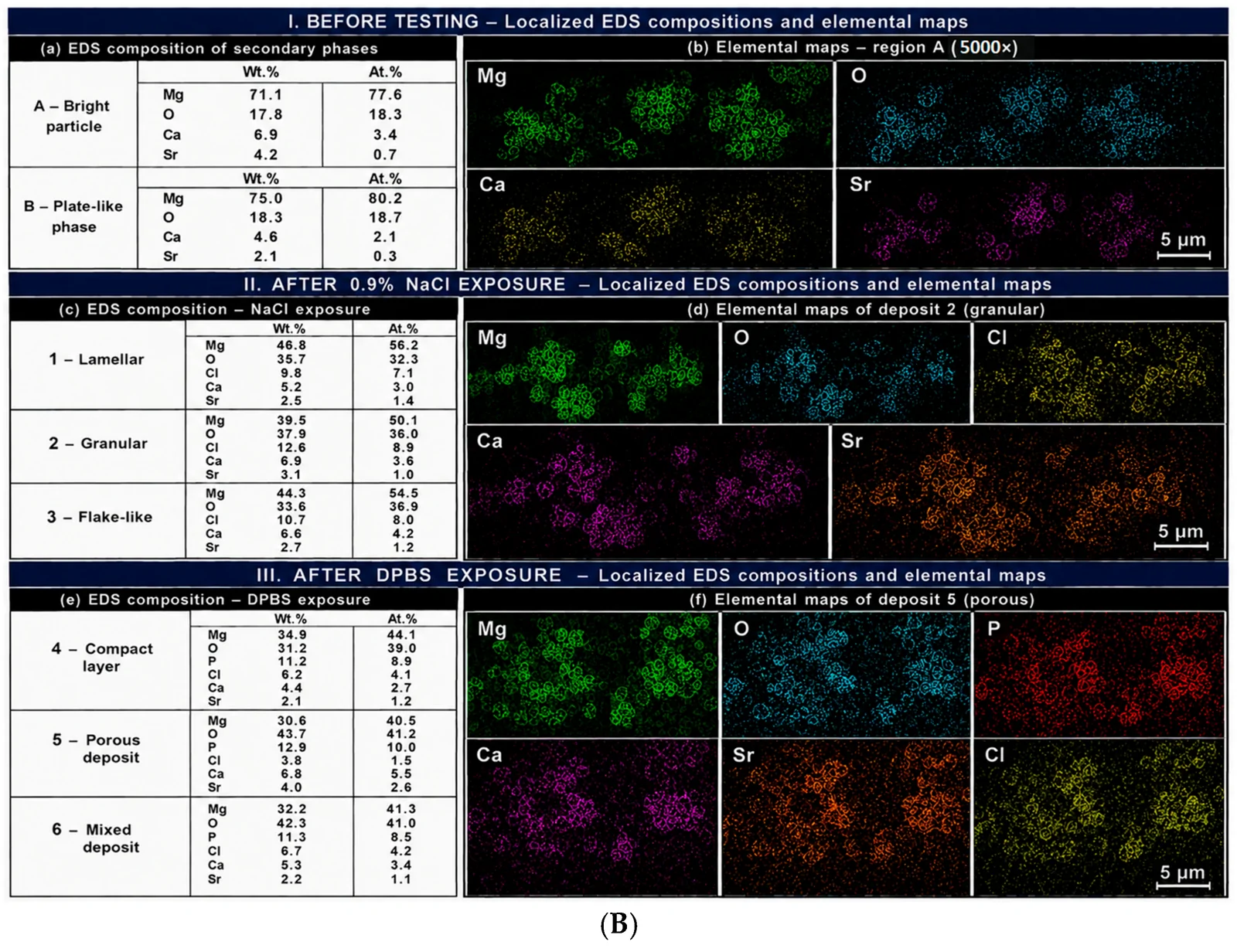
(**A**). Higher-magnification SEM characterization of the Mg–0.5Ca–1.5Sr alloy before testing and after electrochemical exposure. (**a**) Low-magnification overview of the initial surface, with arrows indicating compositionally distinct secondary regions and boxes identifying regions A and B; (**b**,**c**) higher-magnification views of regions A and B, respectively. After exposure to 0.9% NaCl, representative (**d**) lamellar, (**e**) granular, and (**f**) flake-like deposits are shown, with regions 1–3 indicating the sites selected for localized EDS analysis. After exposure to calcium- and magnesium-free Dulbecco’s phosphate-buffered saline (DPBS), representative (**g**) compact cracked, (**h**) porous, and (**i**) mixed granular/plate-like deposits are shown, with regions 4–6 indicating the sites selected for localized EDS analysis. Scale bars are shown in each panel. (**B**). Localized EDS-derived elemental compositions and representative elemental maps of the Mg–0.5Ca–1.5Sr alloy before testing and after electrochemical exposure. (**a**) Localized EDS-derived compositions of the bright particulate region A and the plate-like region B identified on the initial surface; (**b**) representative elemental maps of Mg, O, Ca, and Sr acquired from region A. After exposure to 0.9% NaCl, (**c**) localized EDS-derived compositions of the lamellar, granular, and flake-like deposits identified as regions 1–3, respectively; and (**d**) representative elemental maps of Mg, O, Cl, Ca, and Sr acquired from the granular deposit identified as region 2. After exposure to calcium- and magnesium-free Dulbecco’s phosphate-buffered saline (DPBS), (**e**) localized EDS-derived compositions of the compact, porous, and mixed deposits identified as regions 4–6, respectively; and (**f**) representative elemental maps of Mg, O, P, Cl, Ca, and Sr acquired from the porous deposit identified as region 5. The localized EDS results characterize selected morphological features and should not be interpreted as mean surface compositions. These elemental associations demonstrate regional compositional heterogeneity but do not permit definitive crystallographic or chemical-phase identification. Scale bars are shown in the elemental-map panels.

Higher-magnification SEM examination and localized EDS analysis were performed to characterize the compositionally distinct secondary regions present on the initial Mg–0.5Ca–1.5Sr surface and the morphologically distinct deposits retained after electrochemical exposure. For improved clarity, the results are presented in two separate figures. Figure 5A summarizes the higher-magnification SEM morphology before testing and after exposure to 0.9% NaCl and calcium- and magnesium-free Dulbecco’s phosphate-buffered saline (DPBS), whereas Figure 5B presents the corresponding localized EDS-derived elemental compositions and representative elemental maps.

Before electrochemical testing, the Mg–0.5Ca–1.5Sr surface contained bright particulate and plate-like regions distributed within the matrix and along intergranular or interdendritic areas. These features are indicated by arrows in the low-magnification overview shown in Figure 5A(a), in which regions A and B are also marked for subsequent localized examination. Representative higher-magnification views of these two regions are shown in Figure 5A(b,c). Localized EDS analysis demonstrated differences in the relative Mg, O, Ca, and Sr contents of regions A and B, as summarized in Figure 5B(a), supporting their interpretation as compositionally distinct secondary constituents. Representative elemental maps acquired from region A are presented in Figure 5B(b). Although these localized analyses confirm compositional heterogeneity, the precise crystallographic identity of the secondary phases cannot be established by SEM/EDS alone.

After exposure to 0.9% NaCl, the retained surface products exhibited distinct lamellar, granular, and flake-like morphologies, shown in Figure 5A(d–f). Regions 1–3 mark the corresponding sites selected for localized elemental analysis. The EDS-derived compositions presented in Figure 5B(e) reveal differences in the relative Mg, O, Cl, Ca, and Sr contents among these deposits, indicating marked local compositional heterogeneity. Representative elemental maps acquired from the granular deposit identified as region 2 are shown in Figure 5B(d) and illustrate the spatial distribution of Mg, O, Cl, Ca, and Sr within the analyzed field.

Following exposure to calcium- and magnesium-free DPBS, compact cracked layers, porous network-like deposits, and mixed granular/plate-like aggregates were observed, as shown in Figure 5A(g–i). Regions 4–6 indicate the corresponding sites selected for localized EDS analysis. The localized EDS-derived compositions shown in Figure 5B(e) demonstrate differences in the relative Mg, O, P, Cl, Ca, and Sr contents among these morphologies, further supporting the heterogeneity of the retained surface products. Representative elemental maps acquired from the porous deposit identified as region 5 are presented in Figure 5B(f) and illustrate the spatial distribution of Mg, O, P, Cl, Ca, and Sr within that region.

The localized EDS-derived compositions presented in Figure 5B describe selected morphologically distinct regions and therefore should not be interpreted as mean surface compositions. By contrast, the values reported in Table 6 and Table 7 represent mean ± standard deviation obtained from three independently analyzed surface regions for each alloy–electrolyte condition. Accordingly, these datasets are complementary: Figure 5A documents the local surface morphology, Figure 5B demonstrates localized elemental heterogeneity, and Table 6 and Table 7 summarize the average elemental composition of the analyzed surface deposits.

The EDS-derived semi-quantitative surface elemental compositions expressed as weight percentages are summarized in Table 6. After exposure to 0.9% NaCl, the analyzed surfaces of both alloys were dominated by Mg and O, with detectable Cl and minor Ca and Sr signals. Compared with Mg–0.5Ca–0.5Sr, the Mg–0.5Ca–1.5Sr alloy showed a lower mean Cl content and a higher mean Sr content. After DPBS exposure, P, Na, and K were additionally detected. The Mg–0.5Ca–1.5Sr alloy showed higher mean P and Sr contents and lower mean Cl content than Mg–0.5Ca–0.5Sr, with Sr reaching 1.3 ± 0.2 wt.% compared with 0.2 ± 0.1 wt.%, respectively.

**Table 6:** EDS-derived mean surface elemental composition after electrochemical exposure, expressed as weight percentage (wt.%). Values are presented as mean ± standard deviation from three independently analyzed surface regions per alloy–electrolyte condition. The EDS results are semi-quantitative; oxygen values are included only for comparative surface characterization.

Alloy	Medium	Mg	Na	P	Cl	K	Ca	Sr	O
Mg–0.5Ca–0.5Sr	0.9% NaCl	44.8 ± 1.3	—	—	5.3 ± 0.4	—	0.2 ± 0.1	0.2 ± 0.1	49.5 ± 1.4
Mg–0.5Ca–0.5Sr	DPBS	43.7 ± 1.5	3.6 ± 0.3	3.0 ± 0.3	4.7 ± 0.4	0.3 ± 0.1	0.3 ± 0.1	0.2 ± 0.1	44.2 ± 1.6
Mg–0.5Ca–1.5Sr	0.9% NaCl	41.6 ± 1.5	—	—	2.6 ± 0.3	—	—	0.6 ± 0.1	55.2 ± 1.7
Mg–0.5Ca–1.5Sr	DPBS	45.3 ± 1.6	3.2 ± 0.3	3.8 ± 0.4	2.9 ± 0.3	0.2 ± 0.1	0.4 ± 0.1	1.3 ± 0.2	42.9 ± 1.5

—, not detected in the analyzed regions or below the reporting threshold; DPBS, calcium- and magnesium-free Dulbecco’s phosphate-buffered saline.

The corresponding semi-quantitative atomic-percentage values are presented in Table 7. Oxygen and magnesium were the principal detected elements under both electrolyte conditions. Phosphorus, sodium, and potassium were detected only after DPBS exposure. The chloride atomic percentage was lower on the Mg–0.5Ca–1.5Sr surface than on the Mg–0.5Ca–0.5Sr surface in both media. Sr was also more readily detected on the surface of the alloy containing 1.5 wt.% Sr.

**Table 7:** EDS-derived mean surface elemental composition after electrochemical exposure, expressed as atomic percentage (at.%). Values are presented as mean ± standard deviation from three independently analyzed surface regions per alloy–electrolyte condition. The EDS results are semi-quantitative; oxygen values are included only for comparative surface characterization.

Alloy	Medium	Mg	Na	P	Cl	K	Ca	Sr	O
Mg–0.5Ca–0.5Sr	0.9% NaCl	36.2 ± 1.4	—	—	2.9 ± 0.3	—	0.1 ± 0.1	0.1 ± 0.1	60.7 ± 1.5
Mg–0.5Ca–0.5Sr	DPBS	36.2 ± 1.4	3.2 ± 0.3	1.9 ± 0.2	2.7 ± 0.3	0.2 ± 0.1	0.2 ± 0.1	—	55.6 ± 1.6
Mg–0.5Ca–1.5Sr	0.9% NaCl	32.6 ± 1.5	—	—	1.4 ± 0.2	—	—	0.1 ± 0.1	65.9 ± 1.8
Mg–0.5Ca–1.5Sr	DPBS	38.0 ± 1.4	2.8 ± 0.3	2.5 ± 0.3	1.7 ± 0.2	0.1 ± 0.1	0.2 ± 0.1	0.3 ± 0.1	54.4 ± 1.6

—, not detected in the analyzed regions or below the reporting threshold; DPBS, calcium- and magnesium-free Dulbecco’s phosphate-buffered saline.

**Figure 6:**
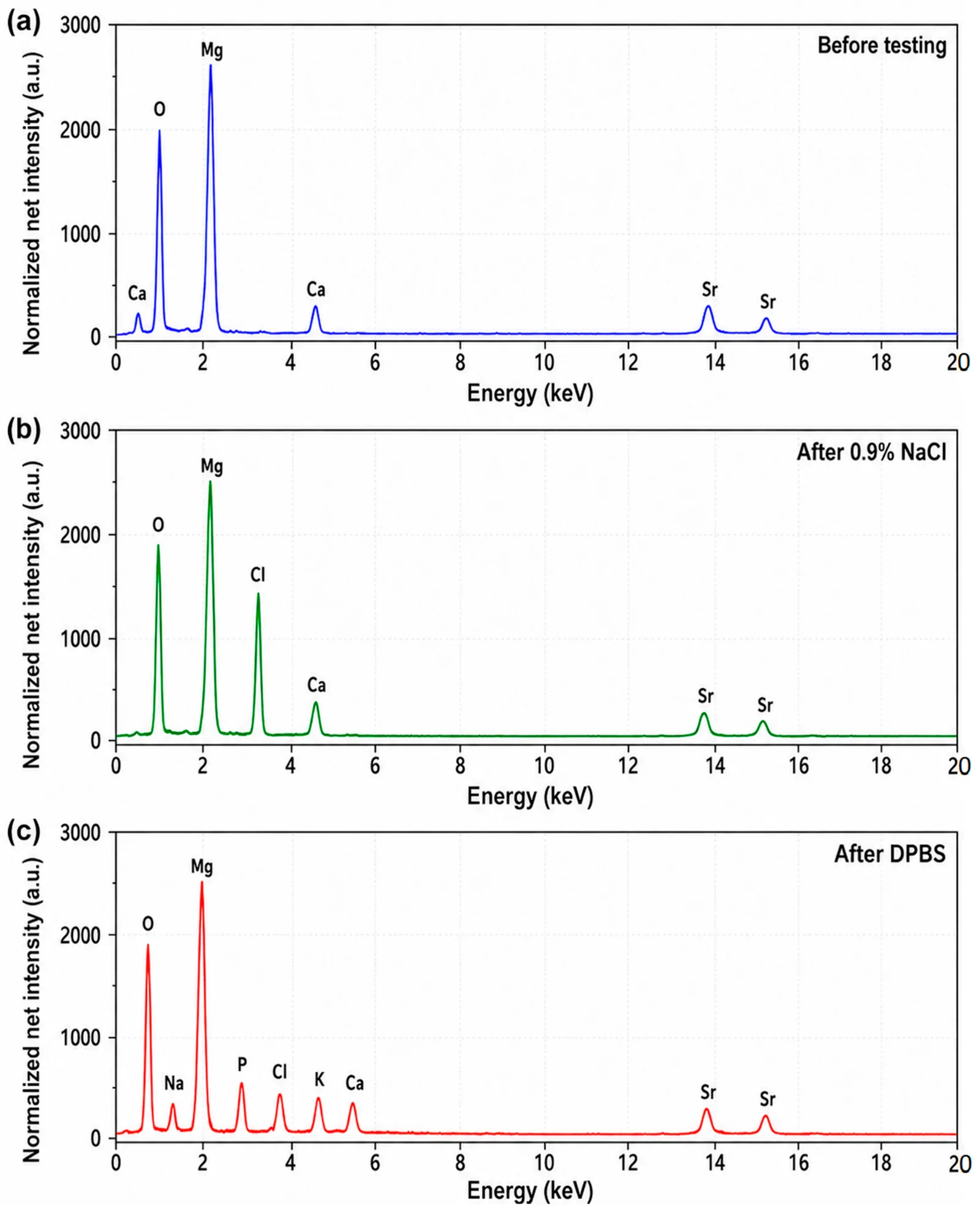
Representative EDS spectra of the Mg–0.5Ca–1.5Sr alloy: (**a**) before electrochemical testing; (**b**) after exposure to 0.9% NaCl; and (**c**) after exposure to calcium- and magnesium-free Dulbecco’s phosphate-buffered saline (DPBS). The spectra were independently normalized to their respective maximum intensities and are displayed over the same energy range for qualitative comparison.

Representative EDS spectra of the Mg–0.5Ca–1.5Sr alloy before electrochemical testing and after exposure to 0.9% NaCl and calcium- and magnesium-free DPBS are presented in Figure 6. Before testing, the spectrum was dominated by Mg, with lower-intensity O, Ca, and Sr signals. After exposure to 0.9% NaCl, Mg and O remained the dominant peaks, together with a detectable Cl signal and minor Ca and Sr signals. After DPBS exposure, additional P, Na, and K signals were detected, together with Mg, O, Cl, Ca, and Sr, consistent with the more complex elemental composition reported in Table 6 and Table 7.

**Figure 7:**
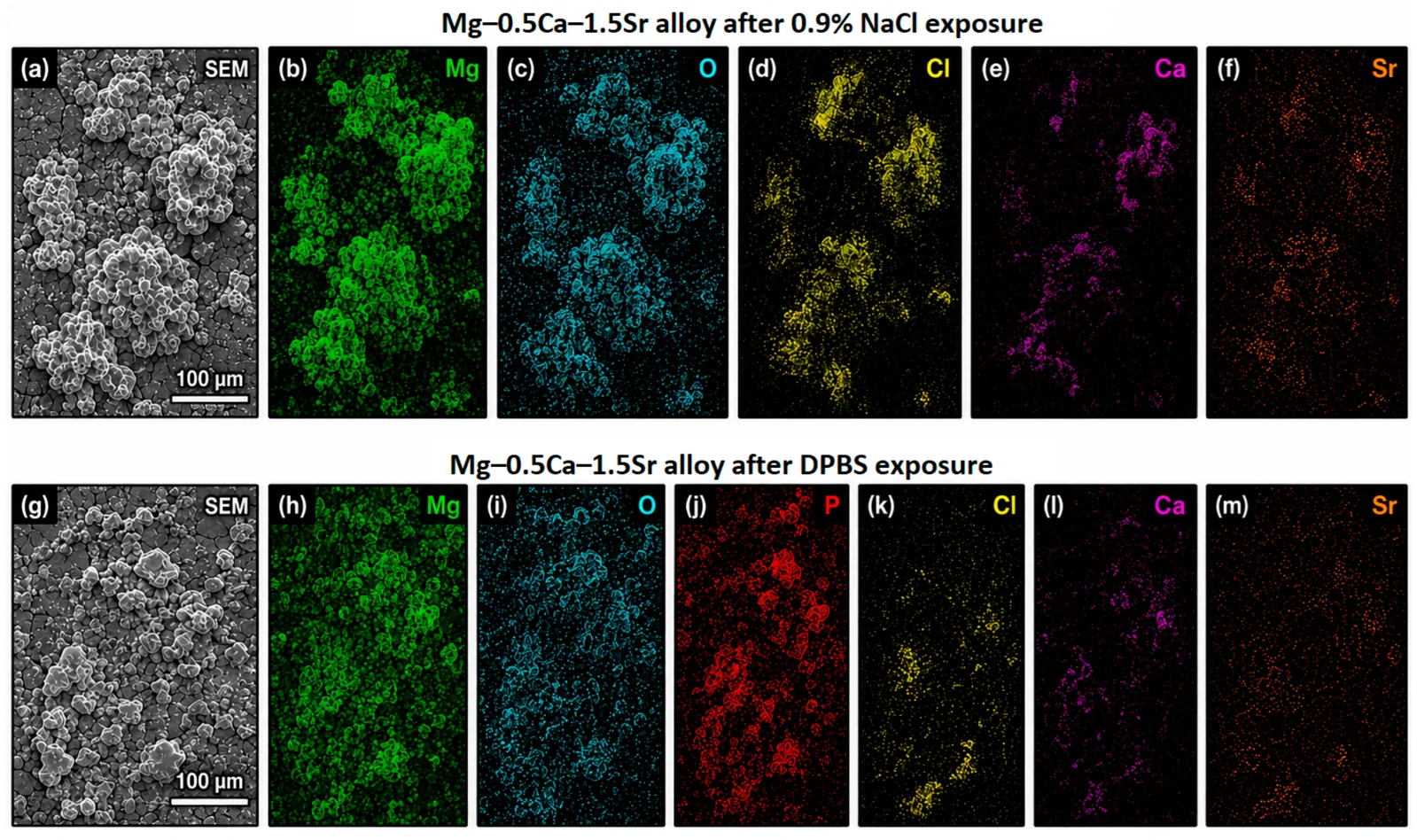
Representative SEM images and corresponding EDS elemental maps of the Mg–0.5Ca–1.5Sr alloy after electrochemical exposure to (**a**–**f**) 0.9% NaCl and (**g**–**m**) calcium- and magnesium-free Dulbecco’s phosphate-buffered saline (DPBS). The mapped elements were Mg, O, Cl, Ca, and Sr after NaCl exposure and Mg, O, P, Cl, Ca, and Sr after DPBS exposure. All elemental maps within each row correspond to the same SEM field. Scale bars: 100 µm.

Representative EDS elemental maps of the Mg–0.5Ca–1.5Sr alloy after exposure to 0.9% NaCl and calcium- and magnesium-free DPBS are presented in Figure 7. After NaCl exposure, Mg and O were broadly distributed across the analyzed surface, whereas Cl was associated predominantly with selected corrosion-product regions. Ca and Sr showed weaker and more localized signals, consistent with their lower surface concentrations. After DPBS exposure, Mg and O remained widely distributed, while P was detected across the retained surface deposits, supporting the presence of P-containing corrosion products. The Cl signal was comparatively less intense and more discontinuous than after NaCl exposure, whereas Ca and Sr remained locally distributed. The elemental maps provide spatial compositional information but do not permit definitive identification of specific crystalline corrosion-product phases.

Overall, the NaCl-exposed regions exhibited Mg-, O-, and Cl-containing surface deposits, whereas the EDS spectra obtained after DPBS exposure indicated a more complex elemental composition that additionally included P, Na, and K. The elemental maps demonstrated electrolyte-dependent differences in the spatial distribution of Mg, O, Cl, Ca, Sr, and, under DPBS conditions, P. These elemental associations are compatible with chloride- and phosphate-containing corrosion products. However, neither EDS spectra nor elemental mapping permits definitive identification of specific crystalline phases or determination of corrosion-product-layer thickness.

## 4. Discussion

### 4.1. Principal Findings

Within the scope of this comparative short-term electrochemical and surface-characterization study, Sr content and electrolyte composition influenced distinct but complementary dimensions of the corrosion response of Mg–0.5Ca–xSr alloys. Increasing Sr content from 0.5 to 1.5 wt.% was associated with lower polarization-derived corrosion-current density and calculated corrosion rate in both electrolytes. This finding supports a more favorable short-term global electrochemical response of the higher-Sr composition under the tested conditions, but does not establish superior long-term degradation behavior or biomedical performance. This Sr-related effect was demonstrated by both the polarization-derived parameters and the replicated EIS measurements. Within each electrolyte, the Mg–0.5Ca–1.5Sr alloy exhibited higher charge-transfer resistance and a more resistive interfacial response than the Mg–0.5Ca–0.5Sr alloy. Thus, EIS independently supported the influence of Sr content on the short-term alloy–electrolyte interfacial response.

Electrolyte chemistry exerted an additional and distinct effect: DPBS produced higher polarization-derived corrosion rates but also a more resistive interface at the open-circuit potential and comparatively continuous P-containing surface deposits. Conversely, NaCl produced lower global corrosion rates during polarization but more porous deposits and a greater number of visible cavity-like defects.

These findings underline the need to integrate electrochemical, morphological, and compositional measurements when evaluating biodegradable magnesium alloys [12,13,14,15,16]. Corrosion-current density, interfacial impedance, visible pit openings, and surface-product chemistry describe related but non-equivalent aspects of degradation [12,13,14,15,16]. This distinction is relevant to biodegradable implants, which must combine controlled material loss with sufficient mechanical retention and an acceptable local biological response [1,3].

### 4.2. Effect of Sr Content on the Electrochemical Response

Within each electrolyte, the Mg–0.5Ca–1.5Sr alloy exhibited lower $j_{corr}$ values and calculated corrosion rates, together with higher $R_{p}$ values, than the Mg–0.5Ca–0.5Sr alloy. Under the present short-term polarization conditions, increasing the Sr content from 0.5 to 1.5 wt.% therefore reduced the global electrochemical dissolution kinetics. The replicated EIS measurements supported this compositional trend at the alloy–electrolyte interface. In 0.9% NaCl, $R_{ct}$ increased from 184 ± 14 Ω·cm^2^ for Mg–0.5Ca–0.5Sr to 253 ± 15 Ω·cm^2^ for Mg–0.5Ca–1.5Sr, whereas in DPBS it increased from 615 ± 42 to 853 ± 48 Ω·cm^2^, respectively. Thus, the higher-Sr alloy exhibited both a more favorable polarization-derived response and a more resistive interface at the stabilized open-circuit potential.

Tafel-derived parameters in magnesium alloys require cautious interpretation because hydrogen evolution, local alkalization, surface-film evolution, dissolution–precipitation processes, and the negative difference effect may alter the polarization response [14,15,16,19]. Accordingly, the reported corrosion-current densities and calculated corrosion rates should be regarded as comparative short-term estimates rather than direct equivalents of long-term degradation determined by immersion, hydrogen-evolution, or gravimetric measurements [14,15,16,19].

Previous studies indicate that Sr content and processing conditions may influence magnesium-alloy corrosion through changes in grain structure, secondary-phase distribution, and electrochemical heterogeneity [6,7,8,9,11,24,25,26]. In the present study, the Mg–0.5Ca–1.5Sr alloy exhibited a more pronounced and comparatively continuous distribution of brighter intergranular or interdendritic regions than the Mg–0.5Ca–0.5Sr alloy. However, because grain size, crystallographic texture, secondary-constituent dimensions and area fraction, and phase identity were not quantified, the present results do not establish a causal microstructural mechanism for the lower polarization-derived dissolution kinetics and higher EIS-derived charge-transfer resistance of the higher-Sr alloy. The findings should therefore be interpreted as specific to the investigated cast microstructures and testing conditions, rather than as evidence that increasing Sr content will invariably improve corrosion resistance under different processing conditions.

### 4.3. Electrolyte- and Sr-Dependent Behavior and Interpretation of EIS

Both alloy compositions exhibited higher polarization-derived corrosion rates in DPBS than in 0.9% NaCl, confirming a strong influence of electrolyte chemistry on the electrochemical response. This effect is likely related to differences in initial pH, buffering capacity, chloride concentration, and phosphate availability. Previous studies have similarly shown that magnesium-alloy degradation depends strongly on the test medium and that electrochemical data should be interpreted together with surface morphology and corrosion-product composition [14,15,16,19,20,21].

The replicated EIS measurements demonstrated effects of both Sr content and electrolyte composition. Within each electrolyte, the Mg–0.5Ca–1.5Sr alloy exhibited larger capacitive loops, higher low-frequency impedance-modulus values, and higher $R_{ct}$ values than Mg–0.5Ca–0.5Sr. In 0.9% NaCl, $R_{ct}$ increased from 184 ± 14 to 253 ± 15 Ω·cm^2^ with increasing Sr content, whereas in DPBS it increased from 615 ± 42 to 853 ± 48 Ω·cm^2^. These findings indicate that increasing the Sr content from 0.5 to 1.5 wt.% was associated with a more resistive alloy–electrolyte interface under both electrolyte conditions.

For both alloys, DPBS produced higher $R_{ct}$ and low-frequency ∣Z∣ values and lower Q values than NaCl. The CPE exponent n remained below unity under all conditions, indicating non-ideal capacitive behavior associated with surface heterogeneity, non-uniform corrosion-product coverage, and spatially distributed interfacial properties. The lower L and higher $R_{L}$ values observed in DPBS further demonstrate that the low-frequency interfacial response differed substantially between the two media. However, the inductive branch remains phenomenological and should not be assigned to a unique adsorption, dissolution, or corrosion-product process without complementary time-dependent electrochemical measurements [14,15,16,19].

The higher $R_{ct}$ and impedance-modulus values measured in DPBS contrasted with the higher polarization-derived corrosion rates observed in that medium. This apparent divergence reflects the different conditions probed by the two electrochemical methods. EIS characterizes the interface under a small-amplitude perturbation near the stabilized open-circuit potential, whereas potentiodynamic polarization progressively displaces the system from this quasi-steady state [14,15,16,19]. Consequently, the higher interfacial resistance measured in DPBS indicates an apparently more resistive interface at the time of measurement but does not demonstrate sustained surface protection or lower cumulative degradation.

The comparatively continuous P-containing deposits observed after DPBS exposure are compatible with partial surface coverage and may have contributed to the higher interfacial impedance. Nevertheless, the higher polarization-derived corrosion rates indicate that these deposits did not provide sustained protection once the system was electrochemically perturbed. The EIS findings should therefore be interpreted together with the polarization, SEM, and EDS results rather than as independent evidence of long-term corrosion resistance [12,13,14,15,16,20,21].

### 4.4. Surface Morphology and Visible Defect Analysis

The NaCl-exposed fields contained more visible defects, higher defect densities, larger affected surface fractions, lower circularity, and higher eccentricity than the corresponding DPBS-exposed fields. These observations indicate that the visible openings remaining after NaCl exposure were more numerous, irregular, and elongated.

However, the lower number of visible cavities after DPBS exposure does not establish that DPBS caused less total degradation. The corrosion products were retained during SEM examination, and the comparatively continuous deposits formed in DPBS may have partially covered or filled surface depressions [12,13]. The image-analysis parameters therefore describe visible pit-like openings in plan view rather than the complete depth, volume, or subsurface geometry of the corrosion damage.

This distinction helps reconcile the morphological results with the higher polarization-derived corrosion rates in DPBS. A surface may undergo substantial electrochemical dissolution while retaining deposits that obscure the underlying damage, whereas discontinuous products may leave more cavities exposed [12,13]. Moreover, because only one representative SEM field was analyzed per condition, the defect measurements should remain descriptive and should not be interpreted as statistically demonstrated differences in pitting susceptibility.

### 4.5. EDS Findings and Surface-Product Interpretation

EDS showed that the NaCl-exposed regions were dominated by Mg, O, and Cl, whereas the DPBS-exposed regions additionally contained P, Na, and K. The Mg–O–Cl elemental association is compatible with oxidized magnesium-containing deposits formed in a chloride-containing electrolyte, whereas the simultaneous presence of Mg, P, and O after DPBS exposure is compatible with phosphate-containing corrosion products [12,13,14,15,16].

These interpretations must remain elemental rather than phase-specific because EDS provides elemental, not crystallographic, information and cannot determine whether the corrosion products are crystalline or amorphous. Consequently, compounds such as Mg(OH)_2_, Mg_3_(PO_4_)_2_, SrHPO_4_, or Sr_3_(PO_4_)_2_ could not be definitively identified by EDS alone [12,13]. Likewise, the higher Sr signal detected on the Mg–0.5Ca–1.5Sr surface, particularly after DPBS exposure, supports local Sr enrichment but does not demonstrate the formation of a discrete Sr-containing or Sr-phosphate phase.

The higher $R_{ct}$ measured in DPBS and the comparatively continuous P-containing deposits are mutually consistent with an apparently more resistive interfacial condition at the time of measurement. Nevertheless, the higher polarization-derived corrosion rate indicates that these deposits did not provide sustained protection against dissolution when the system was electrochemically perturbed.

### 4.6. Biomedical and Processing Implications

The lower global corrosion kinetics of Mg–0.5Ca–1.5Sr may be advantageous for temporary implants that must retain their function during early healing. Mg–Sr and Mg–Ca–Sr alloys have previously supported bone-marrow-derived mesenchymal stem-cell responses, although biological outcomes depend on degradation rate, ion concentrations, and corrosion-product composition [17]. Surface coatings may further regulate the initially rapid degradation of magnesium alloys and improve interfacial stability [18].

Corrosion performance alone is not sufficient to establish biomedical suitability. Sudha et al. evaluated multicomponent magnesium alloys through both biocorrosion and cytotoxicity testing, illustrating the need to relate electrochemical behavior to biological response rather than assuming that a lower corrosion rate automatically ensures better biocompatibility [27].

Processing is also likely to affect the behavior of Mg–Ca–Sr alloys. Yu et al. showed that extrusion-induced microstructural changes altered the corrosion and mechanical properties of a Mg–Zn–Ca–Zr alloy [28]. Accordingly, the present findings apply specifically to the investigated cast microstructures and may differ after thermomechanical processing.

Surface engineering represents another potential optimization route. Li et al. reported that a combined micro-arc oxidation and PLLA coating modified degradation and biological performance in a magnesium alloy [29]. This supports future evaluation of coatings capable of reducing rapid dissolution while preserving controlled Mg and Sr release.

Crystallographic texture may further influence localized and global corrosion independently of nominal composition, as demonstrated for an extruded Mg alloy by Feng et al. [30]. In addition, hot extrusion can modify grain morphology and secondary-constituent distribution, thereby altering the mechanical response of biodegradable magnesium systems [31]. These findings indicate that future optimization should integrate alloy composition, processing, texture, and surface modification rather than considering Sr content in isolation.

### 4.7. Limitations and Overall Interpretation

These limitations define the scope within which the comparative findings should be interpreted. The present study was designed to identify composition- and electrolyte-dependent electrochemical and surface trends under standardized short-term laboratory conditions rather than to reproduce the complexity of physiological degradation. The experiments were performed at 23 ± 1 °C in 0.9% NaCl and calcium- and magnesium-free DPBS. Because temperature and electrolyte composition influence magnesium dissolution, hydrogen evolution, mass transport, and the formation and stability of surface deposits, the absolute electrochemical values should not be extrapolated directly to physiological or in vivo conditions.

An additional limitation is the absence of a Sr-free Mg–0.5Ca reference alloy and of comprehensive quantitative microstructural characterization. Consequently, the present results permit direct comparison between Mg–0.5Ca–0.5Sr and Mg–0.5Ca–1.5Sr but do not distinguish the overall effect of introducing Sr from the effect of increasing its content from 0.5 to 1.5 wt.%. Although SEM/BSE and localized EDS revealed differences in the morphology and distribution of compositionally distinct secondary regions, grain size, crystallographic texture, secondary-constituent dimensions, number density, area fraction, and phase identity were not quantified. Therefore, the specific microstructural mechanism responsible for the different electrochemical responses could not be established. A Mg–0.5Ca control alloy produced under identical conditions, together with quantitative metallography or EBSD and phase identification by XRD and/or TEM, should be included in future studies.

EIS measurements were independently repeated three times for both alloy compositions in both electrolytes, permitting comparison of Sr-content- and electrolyte-dependent impedance responses. Nevertheless, polarization and EIS were performed only after the standardized 30 min open-circuit stabilization period, and no time-resolved EIS, prolonged OCP monitoring, or polarization testing on independent specimens assigned to multiple exposure times was performed. Consequently, the temporal evolution and sustained protective character of the corrosion-product layers could not be established. The temporal stability of the fitted parameters could therefore not be established. Furthermore, although the equivalent-circuit model provided satisfactory fits, its elements remain phenomenological and cannot be assigned uniquely to specific adsorption, dissolution, or corrosion-product processes.

The interpretation of the polarization and surface data is also subject to methodological limitations. Tafel extrapolation in magnesium alloys may be affected by hydrogen evolution, surface-film evolution, local alkalization, and the negative difference effect. Accordingly, the reported corrosion-current densities and calculated corrosion rates should be regarded as method-dependent comparative estimates rather than direct measures of long-term degradation. In addition, the visible-defect analysis was based on one representative SEM field per condition, while SEM/EDS did not determine corrosion-layer thickness, pit depth, phase identity, subsurface damage, hydrogen evolution, ion release, or cumulative mass loss. The quantified defects therefore describe visible pit-like openings within the selected fields and do not establish statistically significant differences in overall pitting susceptibility.

No independent long-term immersion-based degradation experiments were performed under conditions free from externally applied electrochemical perturbation. The present post-exposure SEM/EDS observations were obtained from specimens recovered after approximately 35 min of electrolyte exposure, comprising 30 min of open-circuit stabilization followed by an approximately 5 min potentiodynamic polarization scan. EIS specimens were exposed for approximately 48 min, comprising the 30 min stabilization period followed by an approximately 18 min impedance acquisition. These protocols therefore represent short-term electrochemical testing and are not equivalent to prolonged unperturbed immersion. Gravimetric mass loss, hydrogen evolution, pH evolution, dissolved Mg^2+^ or Sr^2+^ release, post-cleaning surface analysis, and mechanical-property retention were not evaluated. Consequently, the apparent divergence between the higher EIS-derived interfacial resistance and the higher polarization-derived corrosion kinetics observed in DPBS cannot be resolved mechanistically. The higher $R_{ct}$ and impedance-modulus values in DPBS should therefore be interpreted as time-specific interfacial responses near the stabilized open-circuit potential rather than as evidence of sustained surface protection or lower cumulative degradation.

Overall, the hypothesis was partially supported. Increasing the Sr content to 1.5 wt.% was associated with lower polarization-derived global corrosion kinetics and higher EIS-derived charge-transfer resistance in both electrolytes, despite a more pronounced distribution of compositionally distinct secondary regions. DPBS produced a more resistive open-circuit interface and comparatively continuous P-containing deposits but did not prevent higher polarization-derived dissolution, whereas NaCl showed more visible localized defects despite lower global corrosion rates. Thus, Sr content influenced both global corrosion kinetics and interfacial impedance, whereas electrolyte chemistry exerted a strong additional influence on impedance behavior and retained surface morphology. The Mg–0.5Ca–1.5Sr alloy should therefore be regarded as a candidate for further investigation rather than as a material with demonstrated physiological or clinical superiority.

Future studies should include a Sr-free Mg–0.5Ca reference alloy and combine electrochemical characterization with long-term immersion experiments at 37 °C under both static and controlled dynamic conditions. Dynamic conditions should be established using a defined and reproducible flow or agitation protocol. The primary immersion experiments should be performed without potentiodynamic polarization and should incorporate multiple exposure times, gravimetric mass loss, hydrogen evolution, pH evolution, Mg^2+^ and Sr^2+^ release, solution-chemistry monitoring, replicated multi-field surface analysis, post-cleaning and cross-sectional microscopy, and assessment of retained mechanical properties. Future time-resolved protocols should include prolonged OCP monitoring and periodic small-amplitude EIS on dedicated specimens, together with potentiodynamic polarization performed on separate specimens assigned to predefined exposure times, in order to evaluate the formation, stability, and protective character of the corrosion-product layers. Phase characterization using XRD, Raman spectroscopy, FTIR, or XPS should also be included. Additional biologically relevant media, including Kokubo simulated body fluid, Hank’s balanced salt solution, and Ringer’s solution, together with biological testing, will be required before physiological performance or clinical applicability can be inferred.

## 5. Conclusions

This comparative short-term electrochemical and surface-characterization study showed that Sr content and electrolyte composition influenced distinct aspects of the response of cast Mg–0.5Ca–xSr alloys under controlled laboratory conditions. Increasing Sr content from 0.5 to 1.5 wt.% was associated with lower polarization-derived corrosion-current density and calculated corrosion rate and with higher polarization resistance in both 0.9% NaCl and calcium- and magnesium-free DPBS. Replicated EIS measurements additionally showed higher charge-transfer resistance for Mg–0.5Ca–1.5Sr than for Mg–0.5Ca–0.5Sr in both electrolytes. Among the investigated compositions, Mg–0.5Ca–1.5Sr therefore exhibited the more favorable short-term global and interfacial electrochemical response.

Electrolyte composition exerted a separate influence on the alloy–solution interface and retained surface deposits. For both alloy compositions, DPBS produced higher polarization-derived corrosion rates but also higher charge-transfer resistance and higher low-frequency impedance-modulus values at the stabilized open-circuit potential. DPBS additionally produced comparatively continuous P-containing deposits. NaCl produced more porous deposits and a greater number of visible pit-like openings in the analyzed SEM fields. These findings indicate that polarization-derived kinetics, interfacial impedance, and retained-layer morphology describe complementary but non-equivalent aspects of corrosion behavior.

The findings support further investigation of Mg–0.5Ca–1.5Sr as a biodegradable-alloy candidate. However, they represent comparative short-term screening data obtained at 23 ± 1 °C and do not establish long-term degradation behavior, physiological performance, or in vivo suitability. Future studies should evaluate these alloys at 37 °C in additional physiologically relevant media, including Kokubo simulated body fluid, Hank’s balanced salt solution, and Ringer’s solution, with longer immersion periods and time-resolved EIS measurements to assess degradation and surface-layer evolution under conditions more representative of the in vivo environment.

## Figures and Tables

**Table 1:** OES-measured chemical compositions of the experimental Mg–Ca–Sr alloys, including residual trace elements, expressed in wt.%.

Alloy	Mg	Ca	Sr	Fe	Ni	Cu	Mn	Si
Mg–0.5Ca–0.5Sr	98.88	0.52	0.48	0.03	0.01	0.01	0.04	0.03
Mg–0.5Ca–1.5Sr	97.91	0.49	1.52	0.02	0.01	0.01	0.03	0.01

Values are expressed in wt.% and correspond to the OES results obtained for each cast alloy. Minor differences in total composition may occur because of numerical rounding.

**Table 2:** Open-circuit potential evolution of the experimental Mg–Ca–Sr alloys during 30 min immersion in 0.9% NaCl and calcium- and magnesium-free DPBS.

Alloy	Electrolyte	Initial OCP (mV vs. SCE)	OCP at 5 min (mV vs. SCE)	OCP at 10 min (mV vs. SCE)	OCP at 20 min (mV vs. SCE)	OCP at 30 min (mV vs. SCE)	ΔOCP (mV)	Final 5 min Variation (mV)
Mg–0.5Ca–0.5Sr	0.9% NaCl	−1568 ± 9	−1554 ± 8	−1547 ± 7	−1541 ± 6	−1539 ± 6	+29 ± 5	1.6 ± 0.3
Mg–0.5Ca–1.5Sr	0.9% NaCl	−1557 ± 8	−1543 ± 7	−1536 ± 6	−1531 ± 5	−1529 ± 5	+28 ± 4	1.4 ± 0.2
Mg–0.5Ca–0.5Sr	DPBS	−1679 ± 11	−1662 ± 10	−1654 ± 9	−1647 ± 8	−1645 ± 7	+34 ± 6	1.8 ± 0.3
Mg–0.5Ca–1.5Sr	DPBS	−1667 ± 10	−1651 ± 9	−1643 ± 8	−1637 ± 7	−1635 ± 7	+32 ± 5	1.7 ± 0.3

Values are presented as mean ± standard deviation from three independent specimens per alloy–electrolyte condition. ΔOCP was calculated as the OCP at 30 min minus the initial OCP. The final 5 min variation represents the difference between the maximum and minimum OCP values recorded between 25 and 30 min. DPBS, calcium- and magnesium-free Dulbecco’s phosphate-buffered saline; OCP, open-circuit potential; SCE, saturated calomel electrode.

**Table 3:** Electrochemical parameters derived from the potentiodynamic polarization curves of the Mg–Ca–Sr alloys in 0.9% NaCl and DPBS.

Electrolyte	Alloy	$E_{corr}$ (mV vs. SCE)	$j_{corr}$ (mA/cm^2^)	$R_{p}$ (Ω·cm^2^)	$v_{corr}$ (mm/year)	$\beta_{c}$ (mV/dec)	$\beta_{a}$ (mV/dec)
0.9% NaCl	Mg–0.5Ca–0.5Sr	−1421±12	0.0110±0.0014	239±24	2.51±0.32	143±9	199±12
0.9% NaCl	Mg–0.5Ca–1.5Sr	−1414±10	0.0039±0.0005	772±68	0.886±0.110	131±8	213±14
DPBS	Mg–0.5Ca–0.5Sr	−1528±14	0.297±0.026	62±7	6.66±0.58	176±11	92±8
DPBS	Mg–0.5Ca–1.5Sr	−1527±13	0.169±0.018	118±12	3.79±0.41	133±9	136±10

Values are presented as mean ± standard deviation (n=3 independent specimens per alloy–electrolyte combination). $E_{corr}$, corrosion potential; $j_{corr}$, corrosion-current density; $R_{p}$, polarization resistance; $v_{corr}$, calculated corrosion rate; $\beta_{c}$ and $\beta_{a}$, cathodic and anodic Tafel slopes, respectively.

**Table 4:** Equivalent-circuit parameters obtained from EIS fitting for the Mg–0.5Ca–0.5Sr and Mg–0.5Ca–1.5Sr alloys in 0.9% NaCl and DPBS.

Alloy	Electrolyte	R_s_ (Ω·cm^2^)	Q (S·s^n^·cm^−2^)	n	Rct (Ω·cm^2^)	L (H·cm^2^)	R_l_ (Ω·cm^2^)	Reduced χ^2^
Mg–0.5Ca–0.5Sr	0.9% NaCl	75 ± 3	(2.35 ± 0.18) × 10^−5^	0.78 ± 0.03	184 ± 14	2610 ± 210	158 ± 13	(2.7 ± 0.3) × 10^−4^
Mg–0.5Ca–1.5Sr	0.9% NaCl	77 ± 2	(1.79 ± 0.12) × 10^−5^	0.81 ± 0.02	253 ± 15	2298 ± 180	203 ± 17	(2.4 ± 0.2) × 10^−4^
Mg–0.5Ca–0.5Sr	DPBS	12 ± 1	(1.21 ± 0.10) × 10^−6^	0.71 ± 0.03	615 ± 42	58 ± 6	1880 ± 165	(2.0 ± 0.2) × 10^−4^
Mg–0.5Ca–1.5Sr	DPBS	11 ± 1	(8.13 ± 0.65) × 10^−7^	0.75 ± 0.02	853 ± 48	40 ± 4	2571 ± 210	(1.7 ± 0.2) × 10^−4^

$R_{s}$, solution resistance; Q, constant-phase-element coefficient; n, CPE exponent; $R_{ct}$, charge-transfer resistance; L, inductance; $R_{L}$, resistance associated with the inductive branch; DPBS, calcium- and magnesium-free Dulbecco’s phosphate-buffered saline. Values are presented as mean ± standard deviation from three independent specimens per alloy–electrolyte combination. Each EIS spectrum was fitted independently using the equivalent-circuit model described by Equation (3). Reduced $\chi^{2}$ values are reported as mean ± standard deviation and indicate satisfactory agreement between the experimental and fitted spectra.

**Table 5:** Descriptive image-analysis parameters of visible pit-like surface defects after electrochemical exposure. Results refer to one representative SEM field per condition and were not subjected to inferential statistical analysis.

Condition/SEM Field/Predominant Morphology	Detected Defects, n	Density (Defects/mm^2^)	Surface Fraction (%)	Median $d_{eq}$ (µm)	Mean Circularity	Mean Eccentricity
Mg–0.5Ca–0.5Sr/NaCl/Figure 4c/porous–granular	38	1869	0.19	1.02	0.48	0.83
Mg–0.5Ca–0.5Sr/DPBS/Figure 4e/continuous deposits	9	443	0.04	0.66	0.67	0.71
Mg–0.5Ca–1.5Sr/NaCl/Figure 4d/coarse granular	33	1623	0.17	1.02	0.51	0.81
Mg–0.5Ca–1.5Sr/DPBS/Figure 4f/compact–continuous	7	344	0.03	0.61	0.70	0.68

d_eq, equivalent circular diameter of the segmented visible defect; DPBS, calcium- and magnesium-free Dulbecco’s phosphate-buffered saline. The reported values describe only the selected SEM fields identified in Figure 4c–f. Because adherent corrosion products were retained, the segmented features represent visible pit-like openings or surface cavities rather than the complete subsurface geometry of corrosion damage.

## Data Availability

The original contributions presented in this study are included in the article. Further inquiries can be directed to the corresponding authors.

## Supplementary Materials

The following supporting information can be downloaded at: https://www.mdpi.com/article/10.3390/jfb17080418/s1, Table S1: Two-way analysis of variance assessing the effects of Sr content, electrolyte medium, and their interaction on the electrochemical parameters of Mg–Ca–Sr alloys.

## Abbreviations

The following abbreviations are used in this manuscript:

ANOVA	Analysis of variance
BSE	Backscattered electron
CLAHE	Contrast-limited adaptive histogram equalization
CPE	Constant-phase element
DPBS	Dulbecco’s phosphate-buffered saline
EDS	Energy-dispersive X-ray spectroscopy
EIS	Electrochemical impedance spectroscopy
FTIR	Fourier-transform infrared spectroscopy
OCP	Open-circuit potential
PLLA	Poly(L-lactic acid)
rms	Root mean square
SCE	Saturated calomel electrode
SE	Secondary electron
SEM	Scanning electron microscopy
SF_6_	Sulfur hexafluoride
XPS	X-ray photoelectron spectroscopy
XRD	X-ray diffraction
ZAF	Atomic number, absorption, and fluorescence correction
