# Supplementary material for: Comparative Short-Term Electrochemical and Surface Characterization of Biodegradable Mg–Ca–Sr Alloys with Different Strontium Contents
Source: J Funct Biomater. 2026 Aug 20;17(8):418. doi: 10.3390/jfb17080418 (PMC13514709; doi:10.3390/jfb17080418)
Supplement: Supplementary file 1 [file jfb-17-00418-s001.zip › jfb-4477366-supplementary.pdf]

1. Supplementary Table S1

Table S1. Two-way analysis of variance assessing the effects of Sr content, electrolyte medium, and their interaction on the electrochemical parameters of Mg–Ca–Sr alloys.

Outcome	Effect	F	df	p-value
E_{corr}	Sr content	0.315	1, 8	0.5898
E_{corr}	Electrolyte medium	238.424	1, 8	<0.0001
E_{corr}	Sr content × electrolyte	0.177	1, 8	0.6848
j_{corr}	Sr content	54.635	1, 8	<0.0001
j_{corr}	Electrolyte medium	609.127	1, 8	<0.0001
j_{corr}	Sr content × electrolyte	43.754	1, 8	0.0002
R_p	Sr content	192.984	1, 8	<0.0001
R_p	Electrolyte medium	384.143	1, 8	<0.0001
R_p	Sr content × electrolyte	126.569	1, 8	<0.0001
v_{corr}	Sr content	97.881	1, 8	<0.0001
v_{corr}	Electrolyte medium	241.158	1, 8	<0.0001
v_{corr}	Sr content × electrolyte	7.524	1, 8	0.0253

E_{corr}, corrosion potential; j_{corr}, corrosion-current density; R_p, polarization resistance; v_{corr}, calculated corrosion rate. Degrees of freedom are reported as effect df and residual df. Statistical significance was set at $p < 0.05$.
